# *Epimedii Folium* and *Ligustri Lucidi Fructus* Promote Osteoblastogenesis and Inhibit Osteoclastogenesis against Osteoporosis via Acting on Osteoblast-Osteoclast Communication

**DOI:** 10.1155/2023/7212642

**Published:** 2023-01-31

**Authors:** Zitong Ma, Xiufeng Tang, Yuheng Chen, Han Wang, Yuman Li, Yuting Long, Renhui Liu

**Affiliations:** School of Traditional Chinese Medicine, Capital Medical University, Beijing 100069, China

## Abstract

Osteoblast (OB) and osteoclast (OC) play important roles in bone formation and bone resorption, which can communicate with each other through cytokine paracrine. Previous studies have confirmed that *Epimedii Folium* (EF) and *Ligustri Lucidi Fructus* (LLF) used alone or in combination can treat osteoporosis (OP) through regulating bone remodeling, but the effects of EF and LLF on osteoblastogenesis, osteoclastogenesis, and OB-OC communication are unclear. In this study, we investigated the direct and indirect effects of EF and LLF on OBs and OCs via monoculture and coculture (transwell) models of OBs and OCs. We found that the combination of EF and LLF (EF&LLF) could promote osteoblastogenesis and inhibit osteoclastogenesis directly and indirectly. In order to study the mechanisms of EF&LLF on indirectly regulating osteoblastogenesis and osteoclastogenesis, we detected the expression of cytokines by which OBs and OCs could communicate with each other. We found that EF&LLF could downregulate the expression of RANKL and M-CSF and the protein ratio of RANKL/OPG of OBs and Atp6v0d2 expression of OCs and upregulate the expression of OPG and TGF-*β*1 of OBs and the expression of TGF-*β*1, BMP-2, and IGF-1 of OCs, indicating that EF&LLF could regulate cytokine expressions of OBs/OCs to affect OB-OC communication. In addition, EF&LLF had a better effect on regulating cytokines of OBs and OCs than EF or LLF in single use. This study suggested that EF&LLF exhibited the effects of promoting osteoblastogenesis and inhibiting osteoclastogenesis via acting on OB-OC communication and provided some scientific evidences for EF&LLF against OP.

## 1. Introduction

Osteoporosis (OP) is a serious public health problem which is of particular concern in aging society, affecting 23.1% of women and 11.7% of men in the world [1]. OP is characterized by low bone strength and bone mass, leading to an increased risk of fracture [2]. More than half of the osteoporotic fracture patients experience loss of function and require assisted living in the first year. More seriously for the elderly, osteoporotic fracture results in roughly 20% of them dying within one year of fracture [3]. OP seriously affects the physical and mental health of patients and causes large economic and medical burden to the society [4].

Bone is a dynamic tissue remodeled continuously to maintain bone mass and quality—the old or damaged bone is removed by osteoclasts and replaced with new bone formed by osteoblasts [5]. Osteoblast (OB) and osteoclast (OC) as the two main cells participating in this process can communicate with each other through direct cell-cell contact, cytokine paracrine, and cell–bone matrix [6]. Although all three modes of OB-OC communication regulate bone remodeling, we focus on the role of cytokine paracrine in OB-OC communication in this study. OBs and OCs can secrete or influence a range of cytokines to act on another. OBs can secrete nuclear factor kappa B ligand (RANKL), macrophage colony-stimulating factor (M-CSF), and Wnt5A which promote the formation and development of OCs, while OBs also can secrete osteoprotegerin (OPG) and Wnt16 which inhibit OCs. Analogously, OCs can produce tumor necrosis factor *α* (TNF-*α*) during differentiation from macrophage which directly suppress OB differentiation through inhibiting osteogenic factors such as insulin-like growth factor-1 (IGF-1) and runt-related transcription factor 2 (RUNX2) [3]. Besides, OC-mediated bone resorption stimulates the release of transforming growth factor *β*1 (TGF-*β*1), IGF-1, and bone morphogenetic protein-2 (BMP-2) from the bone matrix to promote the differentiation and development of OBs, while OCs secrete d2 isoform of vacuolar (H+) ATPase (Atp6v0d2) at the edge of its resorption comb to inhibit OBs [5].

At present, bisphosphonate, teriparatide, raloxifene, and selective estrogen receptor modulators are the main treatments for OP. However, they are associated with various side effects such as esophagitis and jaw osteonecrosis [7]. Natural products derived from traditional Chinese medicine (TCM) provide alternative options for preventing and treating OP [8]. The formula of *Epimedii Folium* (EF) and *Ligustri Lucidi Fructus* (LLF) is established by Professor *Shizeng Li* for the treatment of OP based on the TCM theory that “the kidney governs the bones”—EF (tonifying kidney-*yang*) and LLF (tonifying kidney-*yin*) take a synergistic effect on tonifying of the kidney. With good curative effect on OP in clinical practice, the combination of EF and LLF (EF&LLF) has been authorized by the State Patent Office of China (Patent No. ZL201410037992.5). Pharmacological studies show that EF, LLF, and their active ingredients (such as icariin and baohuoside I in EF and oleanolic acid and ursolic acid in LLF) have anti-OP effects through improving bone microstructure, increasing bone mineral density, and promoting the differentiation and proliferation of OBs [9–12]. The water extract of LLF can upregulate ER*α* expression of femurs and tibias of ovariectomy- (OVX-) induced OP rats [13]; EF has the estrogen-like effect which is one of the most important mechanisms of EF in treating OP [14]. This may be the mechanism by which EF and LLF synergistically treat OP.

Our previous researches have confirmed that EF&LLF has bone-protective effects by regulating bone remodeling in OVX-induced, natural aging-induced, glucocorticoid-induced, and retinoic acid-induced OP rats [15–18]. However, the effects of EF&LLF on the growth and differentiation of OBs and OCs and their communication at the cellular level are still unclear. This study was aimed at evaluating the effects of EF, LLF, and EF&LLF on osteoblastogenesis and osteoclastogenesis via affecting OB-OC communication in the cell models of OBs and OCs in monoculture and coculture.

## 2. Materials and Methods

### 2.1. Preparation of Medicated Serums

#### 2.1.1. Preparations of Water Extracts

We weighed the appropriate amount of medicinal slice, soaked the medicinal slices with water of 8 times of the volume of medicinal slices for 1 h, then decocted medicinal slices (kept boiling for 30 min on a gentle fire), and filtered the liquid. Then, we decocted the dregs with water of 6 times of the volume of the original medicine (kept boiling for 30 min on a gentle fire) and then filtered. The two decoctions were combined, concentrated, and stored at 4°C. The extraction rates of EF, LLF, and EF&LLF were 0.4 g/mL, 0.3 g/mL, and 0.7 g/mL, respectively.

#### 2.1.2. Animals

32 male Sprague-Dawley rats aged 8 weeks were purchased from Vital River Laboratory Animal Technology Co., Ltd., Beijing, China. The experiment complied with the Animal Management Rule of the Ministry of Public Health, China, and the experimental protocol was approved by the Animal Care Committee of Capital Medical University, Beijing, China. All animals were cared for in the Experimental Animal Center of Capital Medical University. During the whole experiment, the animals were housed at conventional controlled conditions (temperature of 23 ± 2°C, relative humidity of 50 ± 5%, and 12-hour light-dark cycle). They were allowed for free access to standard laboratory food and tap water. All experimental protocols were approved by Ethics Committee of Capital Medical University (No. AEEI-2016-178).

After one week of adaptive feeding, rats were randomly divided into 4 groups (each of 8 rats) and administrated with corresponding drugs by gavage for 7 days, once a day: control group (Co for short, treated with drinking water), EF group (treated with 2 g/kg EF), LLF group (treated with 1.5 g/kg LLF), and EF&LLF group (treated with 2 g/kg EF plus 1.5 g/kg LLF); the gavage volume was 5 mL/kg. One hour after the last administration, we anesthetized rats with 25% ethyl carbamate (4 mL/kg body weight, *i.p.*) and collected abdominal aortic blood of all rats. After placing for 2 h at room temperature, the blood samples were centrifuged at 3000 rpm for 10 min and inactivated at 56°C for 30 min. Then, the serum samples were stored at -20°C after filtering through a micro filtrate membrane (0.22 *μ*m).

### 2.2. Serum Quality Control

We used LC-QQQ-MS/MS (Agilent 6490 series; Santa Clara, CA, USA) for quantitative analysis of 15 components to further detect the active ingredients of all medicated serum. The elution was conducted using Agilent Poroshell 120 EC-C18 column (2.1 × 100 mm and 1.9 *μ*m) at 40°C at a flow rate of 0.3 mL/min, and the mobile phase consisted of 0.1% formic acid (solvent A) and acetonitrile (solvent B). The gradient elution procedures were as follows: 0-1 min at 3% to 8% B, 1-2 min at 8% to 15% B, 2-5 min at 15% to 25% B, 5-6 min at 25% to 30% B, 6-10 min at 30% to 35% B, 10-13 min at 35% to 80% B, 13-18 min at 80% to 95% B, 18-20 min at 95% to 100% B, and 20.01-22 min at 3% B. The injection volume was 2 *μ*L. Mass spectrum parameters are shown in Suppl. table 1. We prepared standard mixtures with methanol, including nuezhenoside G13, oleonuezhenide, epimedin A, epimedin B, epimedin C, ligustroflavone, specneuzhenide, icariin, icaritin, anhydroicaritin, baohuoside I, hyperoside, chlorogenic acid, salidroside, and hydroxytyrosol. Genistein was used as the internal standard. All reference substances were purchased from Pusi Biological Technology Co., Ltd. (Chengdu, China).

100 *μ*L blank serum sample, 10 *μ*L different-concentration standard mixtures, and 200 *μ*L genistein methanol solution with a concentration of 0.20 *μ*g/mL were placed into 1.5 mL EP tubes, mixed, and then centrifuged. The supernatants were dried with nitrogen and redissolved with 100 *μ*L methanol. We detected standard mixtures by LC-QQQ-MS/MS to draw standard curves. Then, we detected drug serum samples and calculated the concentration of 15 components in medicated serum.

### 2.3. Cell Culture

MC3T3-E1 subclone 14 cells (MC3T3-E1 cells, ATCC; Manassas, VA, USA) were cultured in *α*-MEM complete medium (CM) which was constituted by *α*-MEM (Gibco, USA), 10% FBS (Gibco, USA), and 100 U/mL penicillin-streptomycin liquid (PS, Solarbio, Beijing, China), and RAW264.7 cells (Peking Union Cell Resource Center, Beijing, China) were cultured in DMEM CM which was constituted by DMEM (Gibco, USA), 10% FBS, and 100 U/mL PS in a cell incubator (95% air and 5% CO_2_) at 37°C. We replaced the medium every two days and passaged cells when the cells fused to 80%~90%.

The generation of MC3T3-E1 cells used in this study ranged from 15 to 18. The generation of RAW264.7 cells used in this study was less than 15. RAW264.7 cells were induced to differentiate into OCs by treating with 50 ng/mL RANKL (Sino Biological, Beijing, China) for 6 days. We performed alkaline phosphatase (ALP) staining assay on 15-generation MC3T3-E1 cells and tartrate-resistant acid phosphatase (TRAP) staining assay on RANKL-induced RAW264.7 cells. As shown in Suppl. figure 1 A and B, MC3T3-E1 cells used in this study had differentiated into OBs and RAW264.7 cells had differentiated into OCs.

#### 2.3.1. Monoculture

For OB-monoculture experiments, we observed OBs in the normal culture state, TGF-*β*1-induced activation state, and TNF-*α*-induced inhibition state and treated OBs with 2.5% Co serum, 5.0% EF serum, 5.0% LLF serum, or 2.5% EF&LLF serum for 48 h. Then, OBs were performed subsequent tests. For OC-monoculture experiments, we observed OCs in the normal culture state, RANKL-induced activation state, and OPG-induced inhibition state and treated OCs with 15% Co serum, 15% EF serum, 10% LLF serum, and 15% EF&LLF serum for 48 h. Then, OCs were performed subsequent tests. All recombinant proteins were purchased from Sino Biological, Inc. (Beijing, China). The cell grouping and administration methods are shown in Suppl. table 2. The concentrations of medicated serums used in this study were determined by the cell viability tested by MTT; detailed data are shown in Suppl. figure 1.

#### 2.3.2. Coculture

To further study the effects of EF and LLF on OB-OC communication, we established an OB-OC coculture system using 0.4 *μ*m pore transparent polyester membrane 24-well cell culture inserts (Transwell; Corning, USA). RAW264.7 cells pregrown with 50 ng/mL RANKL for 5 days were used to set up the coculture with OBs.

To observe the indirect effects of EF and LLF on OBs, the coculture was set up as follows: RAW264.7 cells were culture in the inserts (200 *μ*L *α*-MEM CM with 50 ng/mL RANKL), and OBs were culture in 24-well (600 *μ*L *α*-MEM CM with 50 ng/mL TNF-*α*). We added medicated serums into the inserts to act on OCs and detected the level of cell viability, apoptosis, proliferation, and ALP activity of OBs. The schematic diagram is shown in suppl. figure 2.

To observe the indirect effects of EF and LLF on OCs, the coculture was set up as follows: OBs were culture in the inserts (200 *μ*L *α*-MEM CM with 50 ng/mL TNF-*α*), and RAW264.7 cells were culture in 24-well (600 *μ*L *α*-MEM CM with 50 ng/mL RANKL). We added medicated serums into the inserts to act on OBs and detected the level of cell viability, apoptosis, proliferation, and the c-terminal type I collagen fragments (CTX) of OCs. The schematic diagram is shown in suppl. figure 3. In these two coculture experiments, cells were cocultivated for 48 h; the detailed methods of cell grouping and administration are shown in Suppl. table 3.

### 2.4. Methyl Thiazolyl Tetrazolium (MTT) Assay

We performed MTT assay to select the optimal serum concentration and detect cell viability. As described previously, MTT solution (5 mg/mL) was added into each well and incubated for 4 h at 37°C [19]. The supernatant was removed, and DMSO was added to dissolve the intracellular crystalline formazan product. Cell viability was determined by measuring the optical density at a wavelength of 490 nm.

### 2.5. Transmission Electron Microscope

OBs and OCs were digested and centrifugated (2000 rpm). After discarding the supernatant, we washed the cells with sterile PBS (15 min × 3 times) and added fetal calf serum. Then, we centrifugated the cells again and discarded supernatant and fixed cells with 4% glutaraldehyde at 4°C overnight. The fixed cell aggregate was cut into 1 mm^3^ blocks, washed with sterile PBS overnight, and fixed with 1% osmic acid for 2 h. Then, cell blocks were dehydrated by gradient with ethanol and acetone, embedded with Epon812, and aggregated at 60°C for 36 h. After being dyed with uranium acetate-lead nitrate, cells were observed under JEM-1400 plus transmission electron microscope.

### 2.6. Cytotoxicity Assay

To evaluate the cytotoxicity of EF and LLF on OBs and OCs, we detected cell viability of OBs and OCs using the WST-1 Cell Proliferation and Cytotoxicity Assay Kit (Beyotime, Shanghai, China), according to the manufacturer's instruction. Briefly, the cells were seeded in 96-well plates (OBs at a density of 5 × 10^3^ cells/well and OCs at a density of 1 × 10^3^ cells/well). After treatment, the cells were incubated with WST-1 solution (20 *μ*L/well) for 2 h; optical density was measured at 450 nm by using SpectraMax iD3 multimode reader (Molecular Devices, USA). The cell viability was expressed as a percentage of the control (the cells cultured normally with CM).

### 2.7. Annexin V-FITC/PI Assay

We used Annexin V-FITC/PI apoptosis kit (Lianke Biotechnology, Hangzhou, China) to detect apoptosis by flow cytometry (FCM). According to the manufacturer's instruction, the main steps were as follows: collecting cells, washing cells with precooling PBS for 2 times and centrifuging it, discarding the supernatant, resuspending cells with 500 *μ*L 1x binding buffer, adding 5 *μ*L Annexin V-FITC and 10 *μ*L PI, incubating cells at room temperature for 5 min in dark place, and finally detecting by BD LSRFortessa customized flow cytometry (BD, USA).

### 2.8. Terminal Deoxynucleotidyl Transferase-Mediated dUTP Nick End Labeling (Tunel) Assay

We used TUNEL BrightGreen Apoptosis Detection Kit (Vazyme, Nanjing, China) to evaluate cell apoptosis. According to manufacturer's instruction, cells were fixed with 4% paraformaldehyde, permeabilized with 0.5% Triton X-100/PBS, incubated with 100 *μ*L equilibration buffer at room temperature for 10 min, incubated with TdT label solution at 37°C for 1 h, washed with PBS, and mounted with antifade mounting medium containing DAPI (Solarbio, Beijing, China). We randomly selected 3 views to calculate mean fluorescence intensity (MFI) of Tunel by Image-Pro Plus 6.0 (Media Cybernetics, USA).

(1) $$\begin{array}{c} \text{MFI}=\frac{\text{sum}-\text{IOD}}{\text{sum}-\text{Area}}. \end{array}$$

### 2.9. EdU Assay

We used BeyoClick™ EdU Cell Proliferation Kit with Alexa Fluor 488 (Beyotime, Shanghai, China) to detect the level of cell proliferation of OBs and OCs, according to manufacturer's instruction. After treatment, cells were incubated with EdU working solution for 2 h. Then, cells were fixed with 4% paraformaldehyde, permeabilized with Immunol Staining Wash Buffer (Beyotime, Shanghai, China), incubated with click additive solution at room temperature for 30 min out of the light, washed with PBS, and incubated with DAPI (Solarbio, Beijing, China). For monoculture cells, we used High Content Screening analyzer (Thermo Scientific, USA) to scan 9 views of one well and calculate the mean of target average intensity (MTAI). For coculture cells, we randomly selected 3 views under Nikon ECLIPSE 80i fluorescence microscope (Nikon, Japan) and calculated MFI of Edu by Image-Pro Plus 6.0.

### 2.10. ALP Testing

We used ALP kit (Nanjing Jiancheng, Nanjing, China) to detect ALP activity of OBs according to the manufacturer's instruction. We detected the change in absorbance at 405 nm at 37°C between the first minute and the third minute and calculated ALP activity relative to control group (cells cultured normally with *α*-MEM CM). We also used the cell ALP stain kit (Nanjing Jiancheng, Nanjing, China) to observe ALP expression in OBs. According to the manufacturer's instruction, we fixed the cells and performed the drip dyeing process. We observed the positive reactions in cell which were characterized as gray-black particles or massive, strip precipitation under Nikon ECLIPSE Ti-U microscope (Nikon, Japan).

### 2.11. TRAP Staining

We used the TRAP stain kit (Solarbio, Beijing, China) to observe osteoclastogenesis. According to the manufacturer's instruction, cells were fixed in stationary liquid for 60 s, stained with TRAP incubated buffer for 60 min at 37°C out of the light, and then redyed with hematoxylin staining solution or methyl green staining solution. We randomly selected 3 views under Nikon ECLIPSE Ti-U microscope. TRAP-positive multinucleated (nuclei > 3) cells were scored as OCs [20]. TRAP-positive multinucleated (nuclei > 10) cells with abundant cytoplasm were scored as mature OCs which had bone absorption capacity [21]. We measured the number of total OCs and the number of mature OCs and calculated the proportion of mature OCs in total OCs.

### 2.12. Bone Resorption Assay

Bone resorption was measured as the formation of resorption pits on bone slices (Nordic Bioscience A/S, DK). The bone slices were washed with *α*-MEM CM for 3 times, and then, RAW264.7 cells were inoculated on bone slices (500 cells/slice). RAW264.7 cells were induced to differentiate into OCs and treated with corresponding drugs. After treatment, the cell culture supernatants were collected for Crosslaps ELISA (BlueGene Biotech, Shanghai, China) to measure the release of the CTX from bone slices. We used OriginPro (OriginLab, USA) to construct the standard curve. The pits on bone slice were visualized by staining with Toluidine Blue O solution (Solarbio, Beijing, China) under Nikon ECLIPSE Ti-U microscope.

### 2.13. Cell Cycle Assay

We used the cell cycle and apoptosis analysis kit (Beyotime Co., Ltd., Shanghai, China) to detect the cell cycle of OBs. According to the manufacturer's instruction, we collected cells and fixed it with precooling 70% ethyl alcohol at 4°C for 2 h. Then, cells were dyed with propidium iodide staining solution at 37°C for 30 min in a dark place and detected by FCM.

### 2.14. Immunofluorescence (IF)

OBs and OCs were fixed with 4% paraformaldehyde and permeabilized with 0.5% Triton X-100 (KeyGEN BioTECH, Jiangsu, China) and then were blocked with 10% normal goat serum (Solarbio, Beijing, China). OBs were incubated with M-CSF, OPG, RANKL, or TGF-*β*1; OCs were incubated with TGF-*β*1, BMP-2, IGF-1, or Atp6v0d2 overnight at 4°C. The description and concentration of primary antibodies used in IF analysis are listed in Suppl. table 4. Then, OBs and OCs were incubated with relevant fluorescent secondary antibodies (KeyGEN BioTECH, Jiangsu, China) at 37°C for 2 h and incubated with DAPI solution (Solarbio, Beijing, China) at room temperature for 5 min. We added antifade mounting medium to the cells and observed the cells under Nikon ECLIPSE 80i fluorescence microscope. We randomly selected 3 views and measured the integral optical density (IOD) by using NIS-Elements BR 3.2 image analysis system (Nikon, Japan).

### 2.15. Quantitative Real-Time PCR (qPCR) Analysis

The mRNA expression was quantified as previously described [22]. We collected OBs and OCs which had been treated with corresponding drugs for 48 h and detected the mRNA expression of M-CSF, OPG, RANKL, and TGF-*β*1 in OBs and the mRNA expression of TGF-*β*1, BMP-2, IGF-1, and Atp6v0d2 in OCs; *β*-actin was used for normalization. The primers used in the qPCR analysis are presented in Suppl. table 5.

### 2.16. Western Blot (WB)

OBs and OCs that had been treated with corresponding drugs for 48 h were collected and lysed by RIPA lysis buffer (including 1%PMSF, 1% protease inhibitor, and 1% phosphatase inhibitor; all reagents used for protein extraction were purchased from New Cell & Molecular Biotech Co., Ltd., Jiangsu, China). The total protein was quantified by bicinchoninic acid (BCA) protein assay kit (Beijing Biosynthesis Biotechnology, Beijing, China). 40 *μ*g total protein in each lane was used for WB analysis as previously described [17]. We examined the expression of TGF-*β*1, M-CSF, OPG, and *β*-tubulin in OBs and TGF-*β*1, ATP6v0d2, BMP-2, and *β*-tubulin in OCs. The description and concentration of primary antibodies used in WB assay are listed in Suppl. table 6. The protein band was visualized by an electro chemiluminescent reagent and exposed to X-film. *β*-Tubulin was used for normalization. The mean density of each protein band was measured by the ImageJ software (National Institutes of Health, USA).

### 2.17. Statistical Analysis

We used SPSS 24.0 (SPSS Inc., Chicago, USA) to perform data analysis. Results of all measurements were presented as means ± standard error (SEM). The statistical differences among groups were evaluated by one-way analysis of variance (ANOVA). The least significant difference (LSD) test when the variances were equal or Tamhane's *T*2 test when the variances were not equal was used for comparisons between individual groups and to determine which means differed significantly statistically (*P* < 0.05).

## 3. Results

### 3.1. Component Content of Medicated Serum

Our previous research has confirmed that EF&LLF can prevent and treat senile OP, postmenopausal OP, glucocorticoid-induced OP, and retinoic acid-induced OP [15–17, 23]. However, we still do not know the physical basis and effective components of EF, LLF, and EF&LLF. In this study, we used LC-QQQ-MS/MS for quantitative analysis of 15 components to determine the physical basis of EF, LLF, and EF&LLF. Regression equation and correlation coefficient *r* of standard curves of 15 components are shown in Suppl. table 7, and the contents of them are shown in Suppl. table 8. The contents of epimedin B, icariin, and salidroside in EF&LLF-medicated serum were significantly higher than that of EF or LLF (all *P* < 0.05), while the content of icaritin in EF-medicated serum was significantly higher than that of EF&LLF (*P* < 0.05).

### 3.2. Indirect Effects of EF&LLF on OBs via OCs

**Figure 1:**
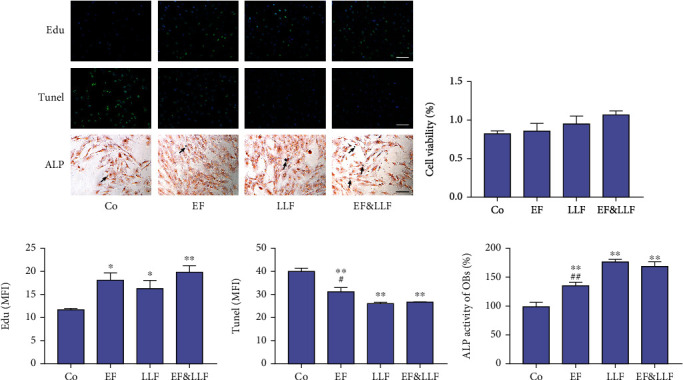
Indirect effects of EF&LLF on OBs via OCs. (a) The representative images of OBs in Edu, Tunel, and ALP assays (scale bars: 100 *μ*m). (b) The cell viability of OBs was measured by MTT assay. The MFIs of (c) Edu and (d) Tunel of OBs were calculated by Image-Pro Plus 6.0. (e) The ALP activity of OBs relative to control group. Data are represented as mean ± SEM, *n* = 3. ^∗^*P* < 0.05 and ^∗∗^*P* < 0.01 compared with the Co group; ^#^*P* < 0.05 and ^##^*P* < 0.01 compared with the EF&LLF group.

In order to observe the indirect effects of EF&LLF on OBs, we established an OB-OC coculture system. We administrated OCs with medicated serums to affect the release of cytokines by OCs which could act on OB by transwell insert and detected the level of proliferation, apoptosis, and differentiation of OBs to observe the indirect effects of EF&LLF on OBs via OCs. During coculture experiment, OBs were treated with TNF-*α* and OCs were treated with RANKL to simulate the cellular states during OP. As shown in Figure 1, all medicated serums could promote OB proliferation and differentiation but inhibit OB apoptosis (*P* < 0.05 or 0.01). The effects of EF&LLF on reducing OB apoptosis and promoting OB differentiation were better than those of EF (*P* < 0.05 or 0.01), indicating that LLF might play a leading role on inhibiting OB apoptosis and promoting OB differentiation in this formula.

### 3.3. Indirect Effects of EF&LLF on OCs via OBs

**Figure 2:**
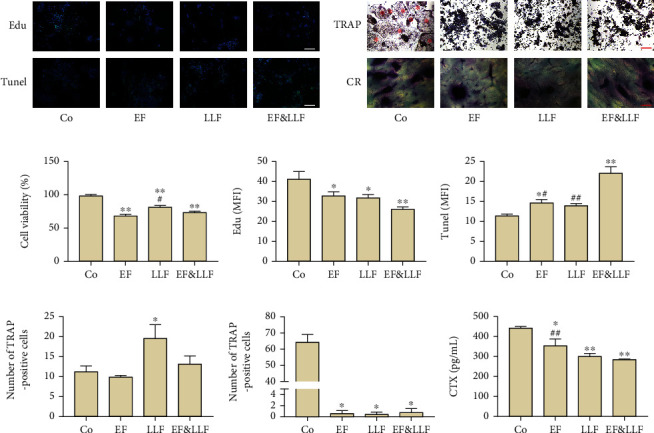
Indirect effects of EF&LLF on OCs via OBs. (a) The representative images of OCs in Edu and Tunel assays (scale bars: 100 *μ*m). (b) The representative images of OCs in TRAP and bone resorption assays (scale bars: 200 *μ*m); the mature OCs were indicated by red asterisks. (c) The cell viability of OCs was measured by MTT assay. The MFIs of (d) Edu and (e) Tunel of OCs were calculated by Image-Pro Plus 6.0. (f) The number of TRAP-positive cells and (g) the proportion of mature OCs in total OCs. (h) The CTX content measured by ELISA assay. Data are represented as mean ± SEM, *n* = 3. ^∗^*P* < 0.05 and ^∗∗^*P* < 0.01 compared with the Co group; ^#^*P* < 0.05 and ^##^*P* < 0.01 compared with the EF&LLF group.

Similarly, in order to observe the indirect effects of EF&LLF on OCs, we administrated OBs with medicated serums to affect the release of cytokines by OBs which could act on OCs by transwell insert and detected the level of proliferation, apoptosis, differentiation, and the bone resorption capacity of OCs. As shown in Figure 2, all medicated serums could inhibit the viability, proliferation, differentiation, maturation, and the bone resorption capacity of OCs (*P* < 0.05 or 0.01). EF and EF&LLF significantly promoted OC apoptosis (*P* < 0.05 or 0.01). LLF notably increased the total number of OCs (*P* < 0.05). The effect of EF&LLF was better than EF and LLF on promoting OC apoptosis, than EF on inhibiting bone resorption (*P* < 0.05 or 0.01), indicating that EF and LLF might take synergistically effect on inhibiting osteoclastogenesis and the bone resorption capacity of OCs.

### 3.4. Effects of EF&LLF on the Cellular State of OBs and OCs

**Figure 3:**
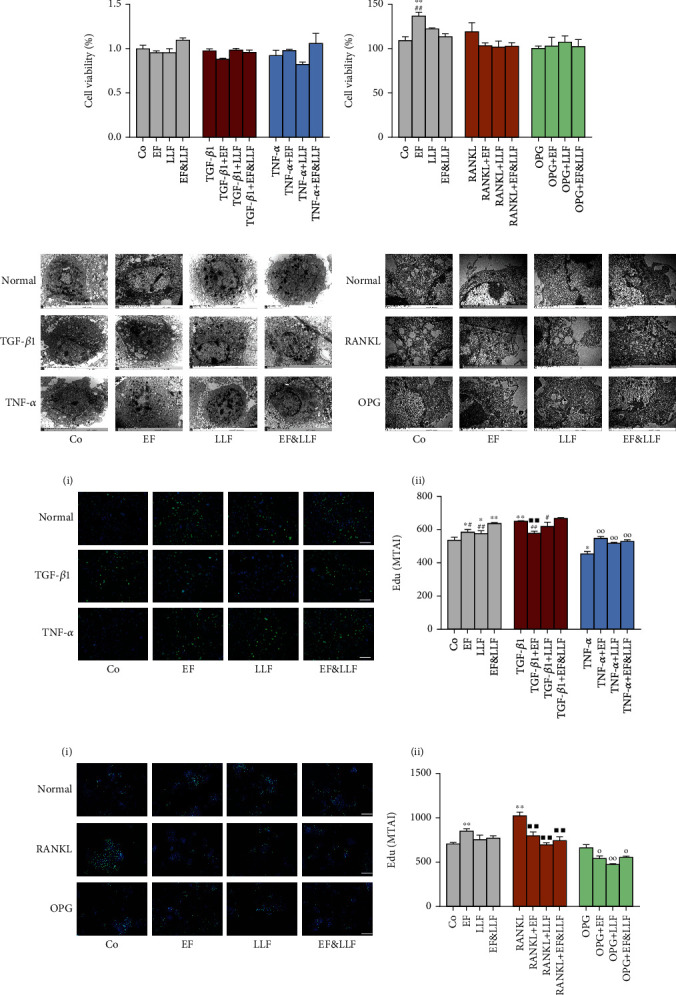
Effects of EF&LLF on cellular state of OBs and OCs. The cell viability of (a) OBs and (b) OCs was measured by WST-1 assay. The ultrastructure of (c) OBs (×10000) and (d) OCs (×30000) was observed by transmission electron microscope. The proliferative activity of (e) OBs and (f) OCs was detected by High Content Screening (scale bars: 200 *μ*m). Data are represented as mean ± SEM, *n* = 3. ^∗^*P* < 0.05 and ^∗∗^*P* < 0.01 compared with the Co group; ^■■^*P* < 0.01 compared with the TGF-*β*1 group in OBs or with the RANKL group in OCs; ^○^*P* < 0.05 or ^○○^*P* < 0.01 compared with the TNF-*α* group in OBs or with the OPG group in OCs; ^#^*P* < 0.05 and ^##^*P* < 0.01 compared with the corresponding EF&LLF group.

We detected the cell viability, ultrastructure, and proliferation activity of OBs and OCs to observe the effects of EF and LLF on the cellular state of OBs and OCs. As shown in Figures 3(a) and 3(b), EF and LLF had no significant toxic effect on OBs and OCs; EF could increase cell viability of OCs cultured normally (*P* < 0.01). The results of transmission electron microscope and EdU assay showed that TGF-*β*1 could markedly promote OB proliferation and enrich organelles of OBs, while TNF-*α* could markedly inhibit OB proliferation and make organelles (such as endoplasmic reticulum and mitochondria) vacuolate (all *P* < 0.01). All medicated serums significantly promoted proliferation of OBs in normal culture and TNF-*α*-induced inhibition states (*P* < 0.05 or 0.01) and increased the number of organelles of OBs in different states. Besides, EF could inhibit proliferation of OBs in TGF-*β*1-induced activation state (*P* < 0.01).

For OCs, we found that RANKL could markedly promote OC proliferation and enrich organelles of OCs (*P* < 0.01); OPG had no effect on OC proliferation but could induce apoptotic morphological changes of OCs. All medicated serums significantly reduced the proliferative activity of OCs in activation and inhibition states (Figure 3(f), *P* < 0.05 or 0.01) decreased the number of organelles of OCs in different states. EF could promote OC proliferation in normal culture state (*P* < 0.01). These results indicated that EF, LLF, and EF&LLF could improve the cellular state of OBs in normal culture and TNF-*α*-induced inhibition states and damaged the cellular state of OCs in RANKL-induced activation and OPG-induced inhibition states; EF might take different regulating effects on the proliferative activity of OBs and OCs when cells were in different states.

### 3.5. Effects of EF&LLF on the Apoptosis Activity of OBs and OCs

**Figure 4:**
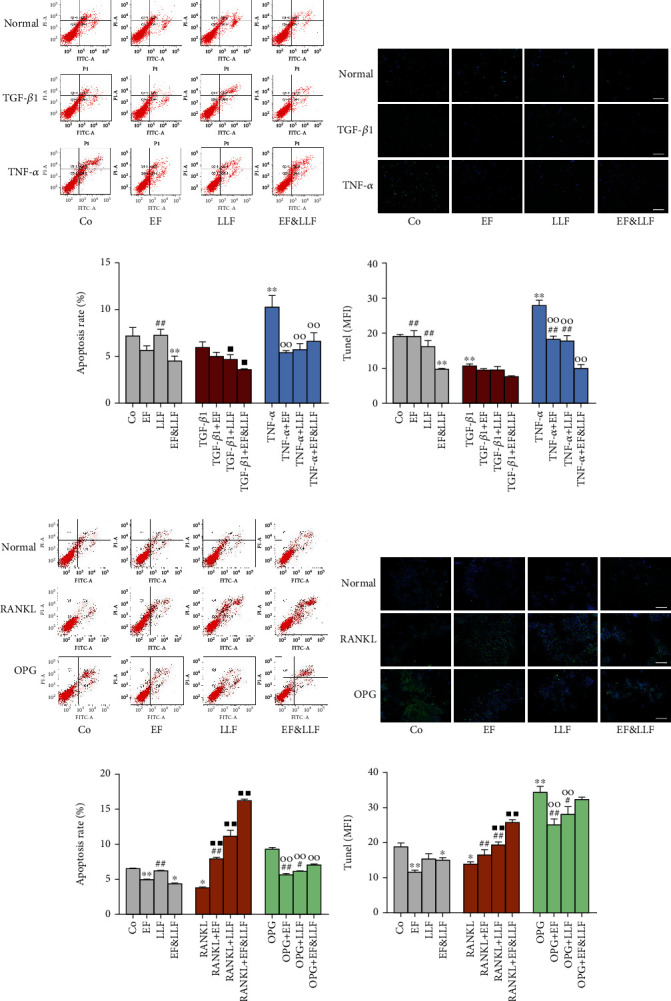
Effects of EF&LLF on apoptosis activity of OBs and OCs. Apoptosis activities of (a–d) OBs and (e–h) OCs were quantified by Annexin V-FITC/PI and Tunel assays (scale bars: 100 *μ*m). Data are represented as mean ± SEM, *n* = 3. ^∗^*P* < 0.05 and ^∗∗^*P* < 0.01 compared with the Co group; ^■^*P* < 0.05 and ^■■^*P* < 0.01 compared with the TGF-*β*1 group in OBs or with the RANKL group in OCs; ^○○^*P* < 0.01 compared with the TNF-*α* group in OBs or with the OPG group in OCs; ^#^*P* < 0.05 and ^##^*P* < 0.01 compared with the corresponding EF&LLF group.

We performed Annexin V-FITC/PI and Tunel assays to evaluate the effects of EF&LLF on apoptosis activity in OBs induced by TGF-*β*1 (inhibiting OB apoptosis) or TNF-*α* (promoting OB apoptosis). All medicated serums could inhibit TNF-*α*-induced OB apoptosis (Figures 4(c) and 4(d), all *P* < 0.01). EF&LLF significantly reduced apoptosis activity of OBs in normal culture and TGF-*β*1-induced activation states (*P* < 0.05 or 0.01). EF&LLF had better effects on inhibiting apoptosis of OBs in normal culture and TNF-*α*-induced inhibition states than EF or LLF (all *P* < 0.01).

The apoptosis activity of OCs was observed as same as OBs. We used RANKL to inhibit OC apoptosis and OPG to promote OC apoptosis. All medicated serums could promote the decreased apoptosis of OCs induced by RANKL but inhibit the increased apoptosis of OCs induced by OPG (Figures 4(g) and 4(h), all *P* < 0.01); EF and EF&LLF markedly inhibited apoptosis of OCs in normal culture state (*P* < 0.05 or 0.01), indicating medicated serums had dual-direction regulation on OC apoptosis. The effect of EF&LLF on promoting RANKL-induced OC apoptosis was better than that of EF or LLF in single use (all *P* < 0.01).

### 3.6. Effects of EF&LLF on the Proliferation and Differentiation of OBs

**Figure 5:**
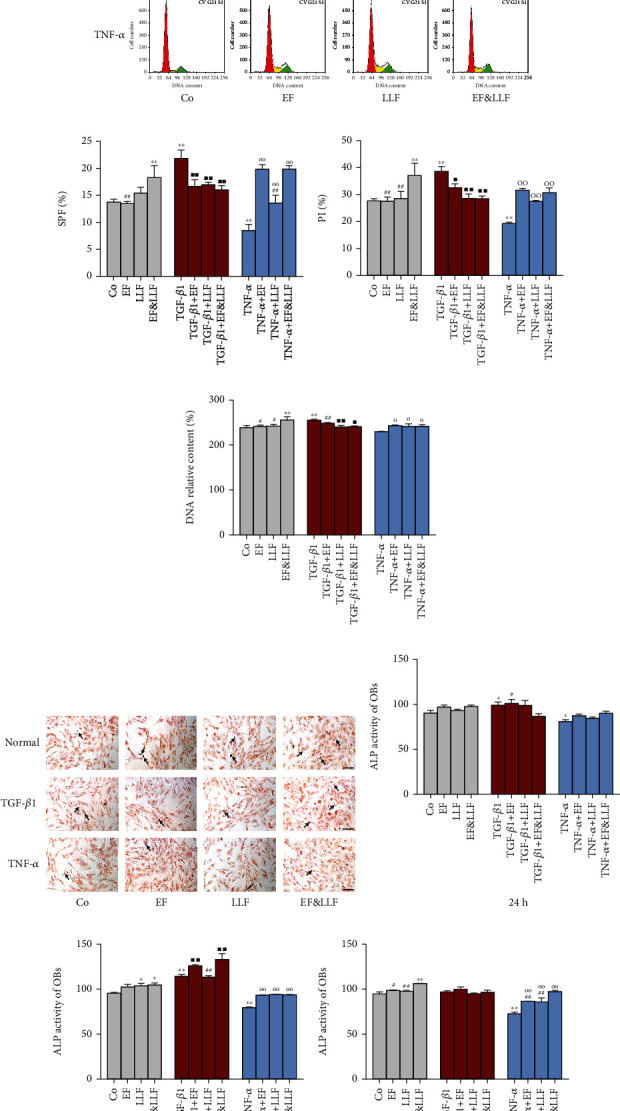
Effects of EF&LLF on the proliferation and differentiation of OBs. (a) The cell cycle distribution of OBs treated for 48 h was detected by FCM and expressed by the proportion of (b) SPF, (c) PI, and (d) DNA relative content. The level of OB differentiation was determined by ALP assay. (e) Representative ALP-staining images of OBs treated for 48 h (scale bars: 100 *μ*m). The ALP activity of OBs treated for (f) 24 h, (g) 48 h, or (h) 72 h. Data are represented as mean ± SEM, *n* = 3. ^∗^*P* < 0.05 and ^∗∗^*P* < 0.01 compared with the Co group; ^■^*P* < 0.05 and ^■■^*P* < 0.01 compared with the TGF-*β*1 group; ^○^*P* < 0.05 and ^○○^*P* < 0.01 compared with the TNF-*α* group; ^#^*P* < 0.05 and ^##^*P* < 0.01 compared with the corresponding EF&LLF group.

We detected the cell cycle of OBs by FCM and calculated the S-phase fraction (SPF), proliferation index (PI), and DNA relative content to evaluate cell proliferation and measured ALP activity of OBs to reflect the level of OB differentiation. As shown in Figure 5, TGF-*β*1 could markedly increase SPF, PI, DNA relative content, and ALP activity (as treating OBs for 24 h or 48 h) of OBs (*P* < 0.05 or 0.01), while TNF-*α* could markedly decrease SPF, PI, and ALP activity of OBs (*P* < 0.05 or 0.01). EF&LLF could increase SPF, PI, and DNA relative content of OBs in normal culture and TNF-*α*-induced inhibition states but decrease those of OBs in TGF-*β*1-induced activation state (*P* < 0.05 or 0.01), indicating that EF&LLF had a dual-direction regulation on OB proliferation. All medicated serums significantly increased ALP activity of OBs which was reduced by TNF-*α* intervention (as treating OBs for 48 h or 72 h, all *P* < 0.01). LLF and EF&LLF could increase ALP activity of OBs cultured normally (as treating OBs for 48 h, *P* < 0.05); EF and EF&LLF could further increase ALP activity of OBs which was already increased by TGF-*β*1 intervention (as treating OBs for 48 h, all *P* < 0.01). The effect of EF&LLF was better than LLF on increasing ALP activity of TGF-*β*1-activated OBs (as treating OBs for 48 h, *P* < 0.01) but was worse than EF (as treating OBs for 24 h, *P* < 0.05) indicating EF played a dominant role in promoting differentiation of OBs in the activated state.

### 3.7. Effects of EF&LLF on the Differentiation and Bone Resorption Capacity of OCs

**Figure 6:**
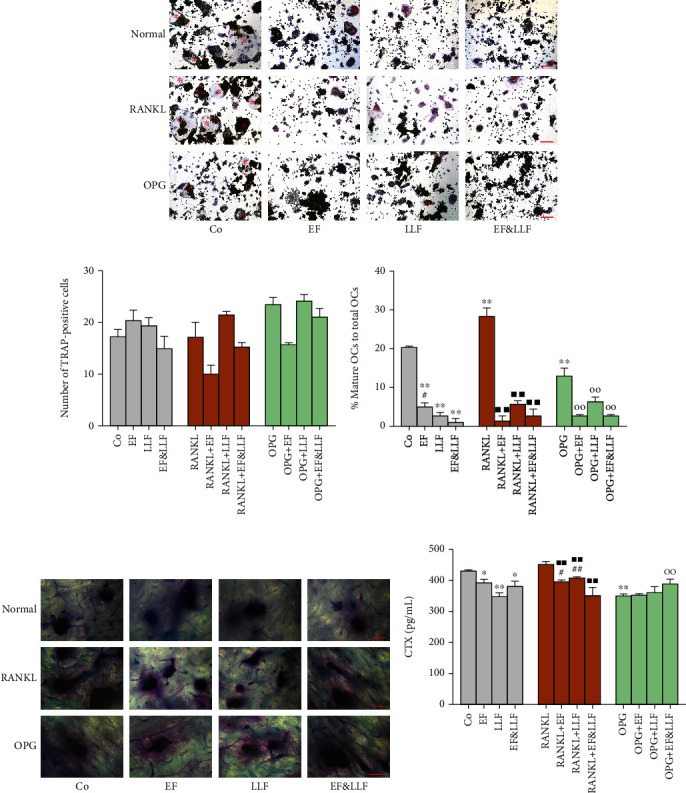
Effects of EF&LLF on differentiation and bone resorption capacity of OCs. (a) The level of OC differentiation was detected by TRAP staining (scale bars: 200 *μ*m) and quantified by (b) the number of TRAP-positive cells and (c) the proportion of mature OCs in total OCs; the mature OCs were indicated by red asterisks. Bone resorption capacity of OCs was evaluated by (d) the pits on bone slice stained by Toluidine Blue O solution (scale bars: 200 *μ*m) and (e) the CTX content measured by ELISA assay. Data are represented as mean ± SEM, *n* = 3. ^∗^*P* < 0.05 and ^∗∗^*P* < 0.01 compared with the Co group; ^■■^*P* < 0.01 compared with the RANKL group; ^○○^*P* < 0.01 compared with the OPG group; ^#^*P* < 0.05 and ^##^*P* < 0.01 compared with the corresponding EF&LLF group.

In order to observe the effects of EF&LLF on OC differentiation, we calculated the number of TRAP-positive cells and the proportion of mature OCs in total OCs. We found that medicated serums had no effect on the total number of OCs but had significant effects on the OC differentiation and mature-OC generation (Figures 6(a)–6(c)). RANKL could markedly promote the differentiation and maturation of OCs, while OPG could inhibit differentiation and maturation of OCs (all *P* < 0.01). All medicated serums could inhibit differentiation and maturation of OCs in different states (all *P* < 0.01). We performed pit formation assay to further study the bone resorption capacity of OCs and found that all medicated serums could inhibit bone resorption capacity of OCs in normal culture and RANKL-induced activation states (*P* < 0.05 or 0.01). But, for the OCs in OPG-induced inhibition state, EF&LLF increased the bone resorption capacity of OCs (*P* < 0.01), which might be related to the inhibitory effect of EF&LLF on OPG-induced OC apoptosis (Figure 4(g)).

### 3.8. Effects of EF&LLF on RANKL/OPG of OBs

During the differentiation process, OBs release lots of cytokines such as M-CSF, RANKL, and OPG which can regulate the growth and differentiation of OCs. RANKL secreted by OBs binds to homologous receptor on the surface of OCs and OC precursors, promoting the differentiation, fusion, and activation of OCs [24]. OPG is the decoy receptor of RANKL which can inhibit OC differentiation and reduce OC count by binding RANKL and blocking RANKL activation. The ratio of RANKL/OPG is considered as a biomarker in bone pathology, which reflects the balance of bone formation and bone resorption [25, 26].

**Figure 7:**
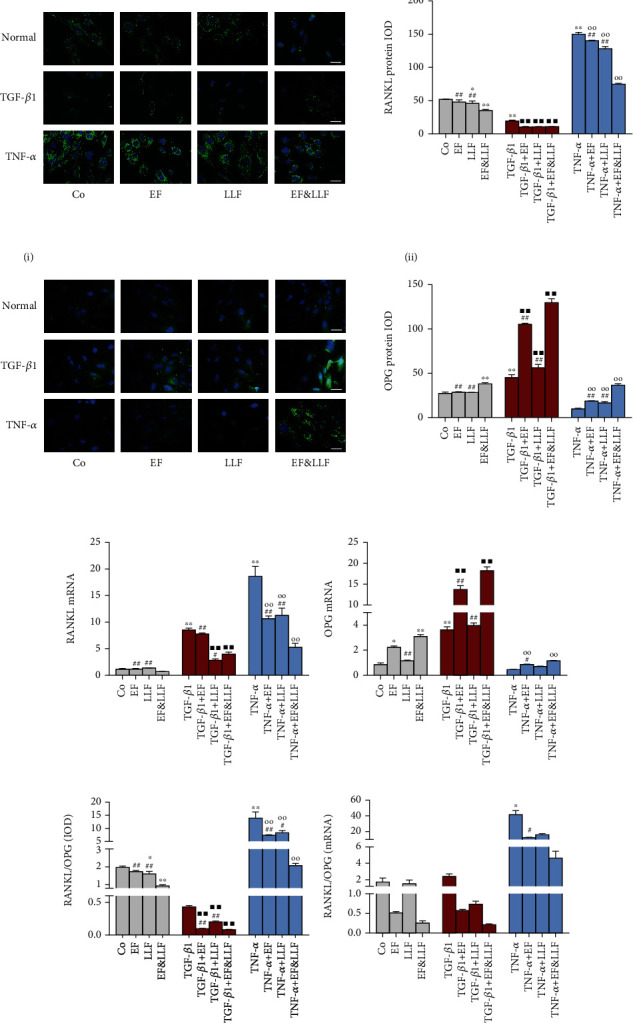
Effects of EF&LLF on RANKL/OPG of OBs. The protein expression of (a) RANKL and (b) OPG of OBs was measured by IF assay (scale bars: 50 *μ*m). The mRNA expression of (c) RANKL and (d) OPG was measured by qPCR analysis with *β*-actin as an internal control. (e) The protein and (f) mRNA ratio of RANKL/OPG were calculated with IF data and qPCR data. Data are represented as mean ± SEM, *n* = 3. ^∗^*P* < 0.05 and ^∗∗^*P* < 0.01 compared with the Co group; ^■■^*P* < 0.01 compared with the TGF-*β*1 group; ^○○^*P* < 0.01 compared with the TNF-*α* group; ^#^*P* < 0.05 and ^##^*P* < 0.01 compared with the corresponding EF&LLF group.

We detected the expression of RANKL and OPG and calculated the ratio of RANKL/OPG. As shown in Figure 7, TGF-*β*1 could decrease RANKL protein expression but increase RANKL mRNA expression and OPG expression in OBs (all *P* < 0.01), while TNF-*α* could increase RANKL expression and the ratio of RANKL/OPG (*P* < 0.05 or 0.01) and reduce OPG protein expression (suppl. figure 4, *P* < 0.01). All medicated serums notably reduced RANKL protein expression and the protein ratio of RANKL/OPG and upregulated OPG protein expression of OBs in both of TGF-*β*1-induced activation and TNF-*α*-induced inhibition states (all *P* < 0.01). For OBs cultured normally, EF&LLF could decrease RANKL protein expression and the protein ratio of RANKL/OPG but increase OPG expression (all *P* < 0.01). EF&LLF had better effects on regulating RANKL and OPG expressions and the protein ratio of RANKL/OPG of OBs in different states than EF or LLF in single use (*P* < 0.05 or 0.01). In addition, EF and EF&LLF could upregulate OPG mRNA expression of OBs in different states (*P* < 0.05 or 0.01), while LLF had no effect on that, indicating that EF might play a leading role in regulating OPG expression of OBs in this formula.

### 3.9. Effects of EF&LLF on M-CSF and TGF-*β*1 of OBs

OBs also influence the differentiation and development of OCs by secreting M-CSF and TGF-*β*1. M-CSF, an essential cytokine of OC differentiation, can regulate the survival, differentiation, and cell migration of OCs by activating the ERK and PI3K/AKT pathways [27]. TGF-*β*1 can regulate bone metabolism by acting on both of OBs and OCs. TGF-*β*1 promotes BMSC migration and OC differentiation through regulating RANKL, M-CSF, and Smads signaling pathways [28].

**Figure 8:**
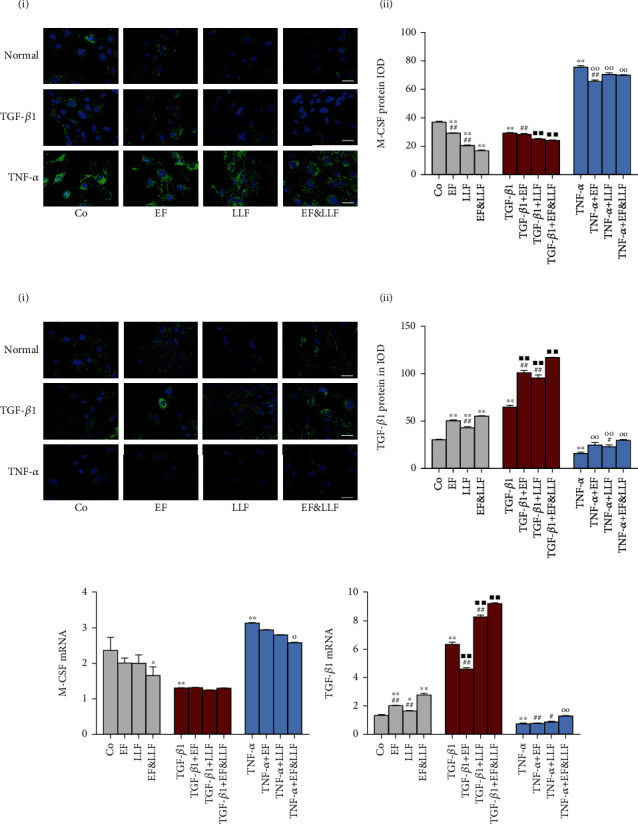
Effects of EF&LLF on M-CSF and TGF-*β*1 of OBs. The protein expression of (a) M-CSF and (b) TGF-*β*1 of OBs was measured by IF assay (scale bars: 50 *μ*m). The mRNA expression of (c) M-CSF and (d) TGF-*β*1 was measured by qPCR analysis with *β*-actin as an internal control. Data are represented as mean ± SEM, *n* = 3. ^∗^*P* < 0.05 and ^∗∗^*P* < 0.01 compared with the Co group; ^■■^*P* < 0.01 compared with the TGF-*β*1 group; ^○^*P* < 0.05 and ^○○^*P* < 0.01 compared with the TNF-*α* group; ^#^*P* < 0.05 and ^##^*P* < 0.01 compared with the corresponding EF&LLF group.

We detected the expression of M-CSF and TGF-*β*1 in OBs and found that TGF-*β*1 markedly decreased M-CSF expression and increased TGF-*β*1 expression (all *P* < 0.01), while TNF-*α* increased M-CSF expression and decreased TGF-*β*1 expression (Figure 8, all *P* < 0.01). All medicated serums significantly reduced M-CSF protein expression of OBs in normal culture and TNF-*α*-induced inhibition states (all *P* < 0.01), increased the protein and mRNA expressions of TGF-*β*1 of OBs in normal culture and TGF-*β*1-induced activation states (*P* < 0.05 or 0.01), and upregulated TGF-*β*1 protein expression of OBs in TNF-*α*-induced inhibition state (all *P* < 0.01). LLF and EF&LLF could reduce M-CSF protein expression of OBs in TGF-*β*1-induced activation state (all *P* < 0.01). EF&LLF also reduced M-CSF mRNA expression of OBs in normal culture and TNF-*α*-induced inhibition states (*P* < 0.05), but EF and LLF had no effect on M-CSF mRNA expression. EF&LLF took better effects than EF on M-CSF protein expression of OBs (all *P* < 0.01), than LLF on TGF-*β*1 expression of OBs in different cellular states (*P* < 0.05 or 0.01), indicating that EF played a major role in upregulating TGF-*β*1 expression and LLF played a major role in downregulating M-CSF expression in this formula.

### 3.10. Effects of EF&LLF on TGF-*β*1 and BMP-2 of OCs

OCs undergo three stages in their differentiation process including hematopoietic stem cells, macrophage colony-forming units, mononucleated OCs, and multinucleated OCs. Bone matrix releases TGF-*β*1 and BMP-2 in response to OC-mediated bone resorption which can act on OB lineage cells to stimulate the differentiation of OBs [29]. TGF-*β*1 can stimulate the proliferation, differentiation, migration, and mineralization of OBs to promote bone repair and bone regeneration [30]. BMP-2 is the key inducer of OB differentiation which can promote bone formation by inducing OB differentiation [31]. In addition, TGF-*β*1 and BMP-2 took a synergistic effect on promoting OB differentiation and maturation [32].

**Figure 9:**
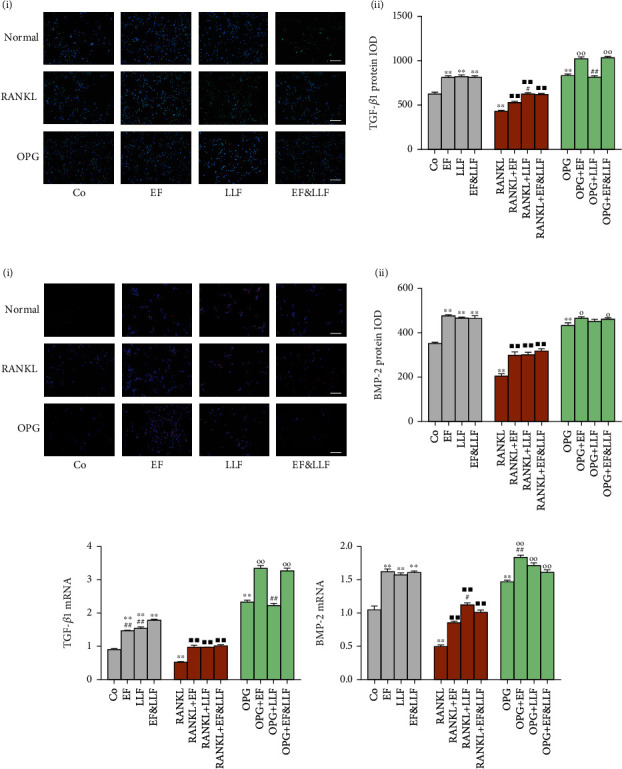
Effects of EF&LLF on TGF-*β*1 and BMP-2 of OCs. The protein expression of (a) TGF-*β*1 and (b) BMP-2 of OCs was measured by IF assay (scale bars: 100 *μ*m). The mRNA expression of (c) TGF-*β*1 and (d) BMP-2 was measured by qPCR analysis with *β*-actin as an internal control. Data are represented as mean ± SEM, *n* = 3. ^∗∗^*P* < 0.01 compared with the Co group; ^■■^*P* < 0.01 compared with the RANKL group; ^○^*P* < 0.05 and ^○○^*P* < 0.01 compared with the OPG group; ^#^*P* < 0.05 and ^##^*P* < 0.01 compared with the corresponding EF&LLF group.

We detected the expression of TGF-*β*1 and BMP-2 in OCs and found that RANKL markedly decreased TGF-*β*1 and BMP-2 expressions of OCs, while OPG decreased TGF-*β*1 and BMP-2 expressions (Figure 9, all *P* < 0.01). All medicated serums significantly upregulated TGF-*β*1 expression of OCs in normal culture and RANKL-induced activation states (all *P* < 0.01) and increased BMP-2 expression of OCs in different states (Figure 9 and suppl. figure 4, *P* < 0.05 or 0.01). EF and EF&LLF significantly increased TGF-*β*1 expression of OCs in OPG-induced inhibition state (all *P* < 0.01). The effect of EF&LLF on increasing TGF-*β*1 expression of OCs in OPG-induced inhibition state was better than that of LLF (all *P* < 0.01). EF took a better effect than EF&LLF on increasing BMP-2 mRNA expression of OCs in OPG-induced inhibition state (*P* < 0.01). LLF took a better effect than EF&LLF on increasing BMP-2 mRNA expression and TGF-*β*1 protein expression of OCs in RANKL-induced activation state (*P* < 0.05). These results indicated that EF and LLF did not take synergistic effect on TGF-*β*1 and BMP-2 expressions of OCs. EF played a major role in acting on OCs in OPG-induced inhibition state while LLF played a major role in RANKL-induced activation state, indicating EF and LLF played complementary roles in acting on OCs.

### 3.11. Effects of EF&LLF on IGF-1 and Atp6v0d2 of OCs

OCs can also regulate the growth and development of OBs by stimulating the secretion of IGF-1 and Atp6v0d2. IGF-1 can promote OB maturation and function, playing a crucial role in postnatal growth; OC-specific IGF1–KO mouse shows growth-restricted phenotype [33]. In addition, OCs can inhibit OB-mediated bone formation by stimulating the secretion of Atp6v0d2 [34].

**Figure 10:**
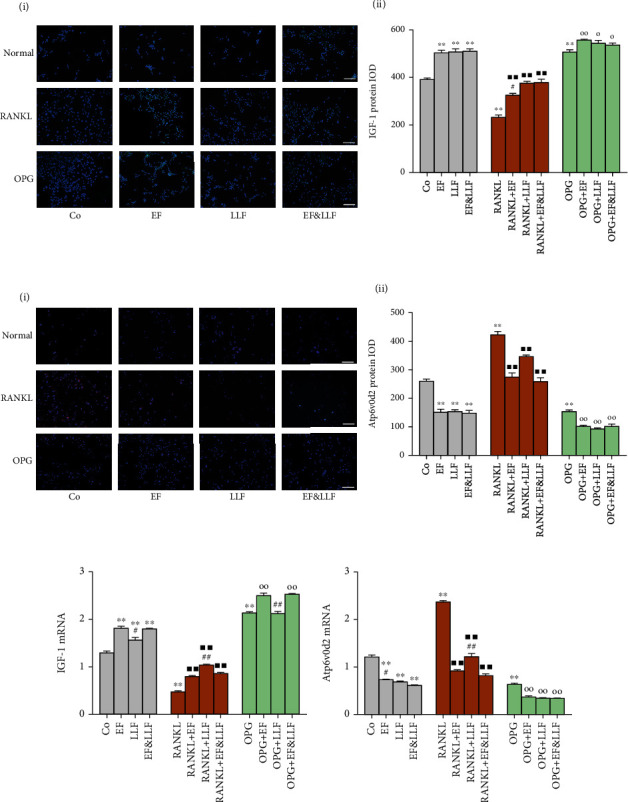
Effects of EF&LLF on IGF-1 and Atp6v0d2 of OCs. The protein expression of (a) IGF-1 and (b) Atp6v0d2 of OCs was measured by IF assay (scale bars: 100 *μ*m). The mRNA expression of (c) IGF-1 and (d) Atp6v0d2 was measured by qPCR analysis with *β*-actin as an internal control. Data are represented as mean ± SEM, *n* = 3. ^∗∗^*P* < 0.01 compared with the Co group; ^■■^*P* < 0.01 compared with the RANKL group; ^○^*P* < 0.05 and ^○○^*P* < 0.01 compared with the OPG group; ^#^*P* < 0.05 and ^##^*P* < 0.01 compared with the corresponding EF&LLF group.

As shown in Figure 10, RANKL markedly decreased IGF-1 expression and increased Atp6v0d2 expression in OCs (all *P* < 0.01), while OPG increased IGF-1 expression and decreased Atp6v0d2 expression (all *P* < 0.01). All medicated serums significantly upregulated IGF protein expression and downregulated Atp6v0d2 expression of OCs in different cellular states (*P* < 0.05 or 0.01) and increased IGF mRNA expression of OCs in normal culture and RANKL-induced activation states (all *P* < 0.01). EF and EF&LLF could increase IGF mRNA expression in OPG-induced inhibition state (all *P* < 0.01). LLF took a better effect than EF&LLF on increasing IGF-1 mRNA expression of OCs in RANKL-induced activation state, while EF&LLF took a better effect than LLF on that of OCs in OPG-induced inhibition state (all *P* < 0.01), indicating that LLF played a major role in upregulating IGF-1 mRNA expression of OCs in activated state and EF played a major role in that of OCs in inhibited state.

## 4. Discussion

OP is a systemic disease characterized by low bone density and increased fragility; over 200 million people suffer from OP around the world [35]. TCM has a long history of treating OP and believes that kidney deficiency and bone marrow loss are the main pathogenesis of OP. The formula of EF and LLF has the efficacy of tonifying gently *yin* and *yang* of the kidney and is commonly used in treating OP. EF has 77 components with the analogous structure to estrogen. Many of these phytoestrogen compounds such as icariin, epimedin A, epimedin B, and epimedin C have osteoprotective effect [36]. Pharmacological studies have shown that EF improves OVX-induced postmenopausal OP rats through increasing the level of serum estradiol and decreasing follicle stimulating hormone [37]. Icariin and baohuoside I can promote the differentiation of bone marrow mesenchymal stem cells (BM-MSCs) into OBs [12, 38]. In addition, icariin can inhibit RANKL-induced osteoclastogenesis via reducing the production of reactive oxygen species and protect the viability and osteogenic potential of OBs via attenuating oxidative stress [39, 40]. Many active ingredients of LLF including tyrosol, hydroxytyrosol, salidroside, oleonuezhenide, and nuezhenoside G13 exhibit antioxidative activity, which is one of the most important mechanisms of LLF in treating OP [41]. Aqueous extract of LLF can improve bone microstructure of OVX-induced postmenopausal OP rats through suppressing oxidative stress response and increasing ER*α* expression in the femurs and tibias [13, 42]. Therefore, antioxidative activity and phytoestrogen property may be the mechanisms by which EF and LLF synergistically treat OP. In previous studies, we have demonstrated that EF&LLF has anti-OP effects on natural aging-induced senile OP, OVX-induced postmenopausal OP, glucocorticoid-induced OP, and retinoic acid-induced OP in animal experiments [15–18]. However, we still do not know the effects of EF&LLF on osteoblastogenesis and osteoclastogenesis at the cellular level. In this study, we observed the direct and indirect effects of EF&LLF on osteoblastogenesis and osteoclastogenesis and explored part of the mechanisms by which EF&LLF regulated OB-OC communication and revealed partial mechanisms of combination of EF and LLF against OP.

### 4.1. Component Content of EF&LLF Medicated Serum

In this study, we detected the content of 15 components in medicated serum to control the serum quality and provide evidence for further experiments about the key active ingredients of EF or LLF on treating OP. We found that the contents of epimedin A, icaritin, and chlorogenic acid were higher in EF-medicated serum; specneuzhenide, salidroside, and hydroxytyrosol were higher in LLF-medicated serum; and icaritin, salidroside, and hydroxytyrosol were higher in EF&LLF-medicated serum. The contents of icaritin, instead of icariin, in EF- and EF&LLF-medicated serums were higher. The possible reason for that might be icariin metabolized into icaritin in vivo [43]. The contents of salidroside and hydroxytyrosol in EF&LLF were higher than those in LLF. Salidroside is one of the main active ingredients of LLF which has good bone-protective effects. Salidroside possesses antiaging, antihypoxia, and antioxidative properties, and takes preventive and therapeutic effects on estrogen deficiency-induced OP through increasing the OPG/RANKL ratio, improving oxidative damage of OBs, and promoting osteogenesis [44–46]; hydroxytyrosol can inhibit OB apoptosis induced by oxidative stress [47]. Therefore, the higher contents of salidroside and hydroxytyrosol in EF&LLF may be the material basis for the better effect of EF&LLF than LLF in treating OP.

### 4.2. The Effects of EF&LLF on OBs in Monoculture

OBs, the bone-forming cells, derive from BM-MSCs which can become OBs, adipocytes, or chondrocytes according to the microenvironment, playing crucial roles in maintaining bone mass and promoting skeletal development [48]. Under the stimulation of transcription factors runt-related transcription factor 2 (RUNX2) and osterix, BM-MSCs differentiate into osteoblastic lineage cells. OB precursors further mature into OBs, following the transcription of early osteogenic genes, such as ALP and collagen1*α*1 chain [49]. Mature OBs have three possible fates: undergoing apoptosis, becoming the bone lining cells, or becoming osteocytes [49]. TGF-*β*1, the most abundant cytokine in bone cells, plays an important role in the process of bone turnover. TGF-*β*1 can stimulate MSC proliferation and promote OB proliferation, differentiation, migration, and mineralization, promoting bone repair and bone regeneration [50, 51]. In addition, TGF-*β*1 can suppress the ability of OBs to secrete RANKL, indirectly inhibiting OC differentiation and affecting bone mass. OCs can stimulate the secretion of TGF-*β*1 from the bone matrix, indicating that the effect of TGF-*β*1 on OBs and OCs is related to OB-OC communication. Suppression of TGF-*β*1 (through TGF-*β* I receptor inhibitors or specific knockout TGF-*β*1) results in decrease in the number of OBs, bone remodeling, trabecular bone parameters, and cortical bone thickness [52]. TNF-*α*, a proinflammatory cytokine, plays an important role in immune and inflammatory responses and promotes osteoclastogenesis and bone resorption by acting on both of OBs and OCs [3]. The monocyte/macrophage lineage precursors can differentiate into OCs and produce a mass of TNF-*α*. TNF-*α* inhibits OB differentiation from BM-MSCs and mineralization by decreasing the expression of RUNX2, IGF-1, and osterix and regulating Wnt, Smads, and NF-*κ*B pathways [3, 53, 54] and indirectly increases osteoclastogenesis by promoting OBs to secrete RANKL and M-CSF [55].

In this study, we used TGF-*β*1 to activate OBs and TNF-*α* to inhibit OBs to make OBs in different states and detected the cell viability, ultrastructure, proliferation, apoptosis, and differentiation of OBs to observe the effects of EF&LLF on OBs in different states. TGF-*β*1 significantly promoted OB proliferation and differentiation and inhibited OB apoptosis; TGF-*β*1-activated OBs had more mitochondria and Golgi apparatus which were OB features. On the contrary, TGF-*β*1 significantly inhibited OB proliferation and differentiation and promoted OB apoptosis; TNF-*α*-inhibited OBs had less organelles, vacuolate endoplasmic reticulum, and mitochondria. EF, LLF, and EF&LLF had no significant toxic effect on OBs. EF&LLF could promote OB differentiation but inhibit apoptosis of OBs in different states and upregulate proliferative activity of OBs in normal culture and TNF-*α*-induced inhibition states but downregulate proliferative activity of OBs in TGF-*β*1-induced activation state, indicating that EF&LLF took a dual-direction regulation on OB proliferation when cells were in different states. In addition, EF&LLF had a better effect on promoting osteoblastogenesis than EF or LLF in single use, indicating that EF and LLF took synergistic effect on promoting osteoblastogenesis.

### 4.3. The Effects of EF&LLF on OCs in Monoculture

OCs, the bone-resorbing cells, derive from hematopoietic stem cells, playing crucial roles in bone resorption. Osteoclastogenesis is mainly stimulated by M-CSF and RANKL; M-CSF participates in the early stage of OC differentiation and promotes the fusion of OC precursors, and RANKL promotes the differentiation of OC precursors into mature OCs [5, 56]. RANKL, TNF ligand superfamily member 11, is also called OPG ligand (OPGL). RANKL secreted by OBs binds to its cognate receptor, receptor activator of NF-*κ*B (RANK), on the surface of OCs and OC precursors. The signal transduction of RANKL/RANK leads to differentiation, fusion, and activation of OCs by activating osteoclastogenic transcription factors (such as NF-*κ*B and activator protein 1) and increasing the expression of osteoclastogenic markers, such as TRAP and Atp6v0d2 [56, 57]. OPG, the TNF receptor superfamily member 11B, is a decoy receptor mainly secreted by OBs and bone marrow stromal cells. The affinity of OPG to bind RANKL is 500-fold higher than that of RANK. Through competitive binding with RANKL, OPG inhibits osteoclastogenesis and OC-mediated bone resorption [58].

In this study, we used RANKL to activate OCs and OPG to inhibit OCs to make OCs in different states and detected the cell viability, ultrastructure, proliferation, apoptosis, differentiation, and bone resorption capacity of OCs to observe the effects of EF&LLF on OCs in different states. RANKL significantly promoted OC proliferation and differentiation, inhibited OC apoptosis, and improved ultrastructure of OCs. On the contrary, OPG significantly promoted OC apoptosis, inhibited differentiation and bone resorption capacity of OCs, and induced ultrastructure of OCs showed apoptotic morphological changes. EF, LLF, and EF&LLF had no significant toxic effect on OCs but injured OC ultrastructure. EF&LLF could inhibit differentiation of OCs in different states, reduce proliferative activity of OCs in RANKL-induced activation and OPG-induced inhibition states, inhibit apoptosis of OCs in normal culture and OPG-induced inhibition states but promote apoptosis of OCs in RANKL-induced activation state and downregulate bone resorption capacity of OCs in normal culture and RANKL-induced activation states but upregulate bone resorption capacity of OCs in OPG-induced inhibition state, indicating EF&LLF took a dual-direction regulation on apoptosis and bone resorption capacity of OCs.

### 4.4. The Indirect Effects of EF&LLF on OBs and OCs

The study of in vitro OB-OC cocultures started in 1982. According to the purpose of research, the method of establishment of OB-OC coculture is different [59]. In this study, we focused on OB-OC communication via cytokine paracrine. Hence, we chose the 0.4 *μ*m pore transparent polyester membrane between the two chambers of the transwell which allowed for the OBs and OCs to communicate via passage of factors released into the media [60]. We took differentiated-OBs and differentiated-OCs as research objects and treated OBs with TNF-*α* and treated OCs with RANKL to simulate the celluar state under OP pathology. We observed the cell viability, proliferative activity, apoptosis activity, and the level of differentiation of OBs/OCs as treating OCs/OBs with medicated serums. EF, LLF, and EF&LLF could promote OB proliferation and differentiation but inhibit OB apoptosis via acting on OCs; EF&LLF had better effects than EF on affecting OB apoptosis and differentiation, indicating LLF played the major role in regulating OB apoptosis and differentiation via acting on OCs in the formula of EF and LLF. Similarly, EF, LLF, and EF&LLF could reduce the level of cell viability, proliferative activity, maturation, and the bone resorption capacity of OCs through acting on OBs. EF and LLF could promote OC apoptosis. These results suggested that EF&LLF could promote osteoblastogenesis and inhibit osteoclastogenesis through acting on OB-OC communication.

### 4.5. Effect of EF&LLF on Cytokines Regulating OCs in OBs in Different States

OB-OC communication controls bone remodeling throughout life through direct cell-cell contact, cytokines, and extracellular matrix interactions [61]. OBs can secrete M-CSF, RANKL, OPG, and TGF-*β*1 to affect OC formation, differentiation, and apoptosis [34, 50]. M-CSF is an essential cytokine in the survival, differentiation, and migration of OCs, upregulating RANK expression in bone marrow precursors and inducing bone marrow precursors to differentiate into OC precursors [34]. Estrogen deficiency promotes M-CSF secretion in OBs to enhance osteoclastogenesis [62]. RANKL stimulates OC precursors differentiate into OCs by binding to RANK located in the cell membrane of OC precursor, while OPG can prevent RANK-RANKL binding by binding to RANKL to inhibit osteoclastogenesis [63]. The ratio of RANKL/OPG is usually used in reflecting the ability of osteoclastogenesis and the degree of OC-mediated bone resorption. A high RANKL/OPG ratio reflects an increase in bone loss, while a low ratio reflects a decrease in bone loss [64]. OBs also secrete a mass of TGF-*β*1 considered as the core cytokine that dominate bone formation. However, TGF-*β*1 has dual-direction regulation on OC differentiation and development. On the one hand, TGF-*β*1 decreases RANKL expression and increases OPG expression in OBs, resulting in indirectly inhibiting OC formation. On the other hand, TGF-*β*1 can directly stimulate RANKL to promote OC differentiation and cooperate with TNF-*α* to promote OC differentiation independent of RANKL signaling [63].

In order to study the mechanism of EF&LLF on regulating OCs via OB-OC communication, we detected the expression of cytokines which could regulate OCs in OBs. We used TGF-*β*1 and TNF-*α* to treat OBs to establish the cell model of OBs in activation and inhibition states and detected the expression of M-CSF, RANKL, OPG, and TGF-*β*1 in OBs. EF&LLF could decrease the expression of RANKL and M-CSF and the protein ratio of RANKL/OPG and increase the expression of OPG and TGF-*β*1 of OBs in normal, activated, and inhibited states, indicating that the effect of EF&LLF on inhibiting indirectly osteoclastogenesis might be related to acting on the cytokine expressions of OBs.

### 4.6. Effect of EF&LLF on Cytokines Regulating OBs in OCs in Different States

OB-OC communication takes part in the control of bone homeostasis and disruption of which can lead to metabolic bone diseases including OP and osteopetrosis [65]. Same as the effect of OBs on OCs, OCs can influence the function of OBs through regulating cytokines such as TGF-*β*1, BMP-2, IGF-1, and Atp6v0d2. TGF-*β*1 is released from the bone matrix due to OC-mediated bone resorption and is activated in acidic environment resulting from activated OCs [64]. TGF-*β*1 promotes BM-MSC proliferation, migration, and differentiation into OBs through Smad2/3 pathway and promotes OB proliferation and matrix maturation [66, 67]. BMP-2 plays an important role in bone remodeling and homeostasis, promoting OB differentiation through activating osteogenic genes including RUNX2 (which stimulates MSCs differentiate into OBs) and osterix (which stimulates OB precursors to differentiate into mature OBs and form bone) [68, 69]. IGF-1, an anabolic hormone, can enhance OB function and stimulate OB proliferation and differentiation by binding to specific membrane receptors of OBs; IGF-1 can also regulate bone mass, quality, and mineralization through increasing collagen synthesis and decreasing its degradation [70]. Atp6v0d2 is highly expressed in OCs which promotes OC maturation (not differentiation) [5]. However, the exact effect of Atp6v0d2 on OBs and OCs is unclear. It has been observed that the OB function, bone formation, and bone mass were significantly increased in Atp6v0d2-deficient mice, which may be due to OB-extrinsic factors [71].

In order to study the mechanism of EF&LLF on regulating OBs via OB-OC communication, we detected the expression of cytokines which could regulate OBs in OCs. We used RANKL and OPG to treat OCs to establish the cell model of OCs in activation and inhibition states and detected the expression of TGF-*β*1, BMP-2, IGF-1, and Atp6v0d2 in OCs. EF&LLF could increase the expression of TGF-*β*1, BMP-2, and IGF-1 but decrease Atp6v0d2 expression of OCs in normal, activated, and inhibited states, indicating that the effect of EF&LLF on promoting indirectly osteoblastogenesis might be related to acting on the cytokine expressions of OCs.

### 4.7. Limitations of This Study

In this study, we confirmed that EF&LLF could promote osteoblastogenesis and inhibit osteoclastogenesis via regulating the cytokine paracrine in OB-OC communication. Except the cytokine paracrine, OB and OC can communicate with each other through direct cell contact and cell–bone matrix. Therefore, we need other OB-OC coculture models such as the layered coculture model to observe the effect of EF&LLF on OB-OC direct cell contact [60]. In addition, bone remodeling is mediated by the basic multicellular unit which is composed of OBs, OCs, the osteocytes within the bone matrix, and the bone lining cells. Except OB-OC communication, osteoblastogenesis and osteoclastogenesis are also affected by osteocytes, bone lining cells, and vascular endothelial cells [72]. We need more experiments to investigate whether EF&LLF can affect osteoblastogenesis and osteoclastogenesis via other cell types.

## 5. Conclusions

In summary, EF&LLF could promote OB proliferation and differentiation and inhibit OB apoptosis via acting on OCs; reduce the cell viability, proliferation, differentiation, and bone resorption capacity of OCs and promote OC apoptosis via acting on OBs. EF&LLF could decrease RANKL and M-CSF expressions and the protein ratio of RANKL/OPG of OBs and Atp6v0d2 expression of OCs and increase OPG and TGF-*β*1 expressions of OBs and TGF-*β*1, BMP-2, and IGF-1 expressions of OCs, indicating that the effect of EF&LLF on indirectly promoting osteoblastogenesis and inhibiting osteoclastogenesis might be related to regulating the cytokine paracrine in OB-OC communication. In addition, EF&LLF took better effects on regulating the expression of cytokines and the apoptosis activity of OBs and OCs, inhibiting bone resorption capacity of OCs and promoting OB differentiation than EF or LLF, indicating that combination of EF and LLF had better effects in the prevention and treatment of OP than EF or LLF in single use.

## Data Availability

The data used and/or analyzed during the current study are available from the corresponding author on reasonable request.

## Supplementary Materials

Supplementary Materials Suppl. table 1: the working parameters of 15 components by LC-MS/MS. Suppl. Figure 1: cell identification and determination of medicated serum concentrations by MTT assay. (A) Representative ALP-staining image of OBs (scale bar: 100 *μ*m). (B) Representative TRAP staining image of OCs (scale bar: 100 *μ*m). The cell viability of (C) OBs and (D) OCs which were cultured for 24 h or 48 h with different concentrations of (i) Co serum, (ii) EF serum, (iii) LLF serum, and (iv) EF&LLF serum. Data are represented as mean ± SEM, *n* = 3. ^∗^*P* < 0.05 and ^∗∗^*P* < 0.01 compared with the serum-free group. Suppl. table 2: the methods of cell grouping and administration. Suppl. Figure 2: the schematic diagram of OB-OC coculture by which we observed the indirect effect of EF&LLF on OBs. We created this schematic diagram with http://biorender.com. Suppl. Figure 3: the schematic diagram of OB-OC coculture by which we observed the indirect effect of EF&LLF on OCs. We created this schematic diagram with http://biorender.com. Suppl. table 3: the administration methods of coculture system. Suppl. table 4: the antibodies used for IF analysis. Suppl. table 5: the primers used for qPCR analysis. Suppl. table 6: the antibodies used for WB analysis. Suppl. table 7: regression equation and correlation coefficient *r* of standard curves of 15 components. Suppl. table 8: components content of medicated serum. Suppl. Figure 4: the cytokine expressions of OBs and OCs detected by WB. (A–D) The protein expression of TGF-*β*1, M-CSF, and OPG of OBs was measured by WB assay, and *β*-tubulin was used for normalization. (E–H) The protein expression of TGF-*β*1, Atp6v0d2, and BMP-2 of OCs was measured by WB assay, and *β*-tubulin was used for normalization.
