# Supplementary material for: Epimedii Folium and Ligustri Lucidi Fructus Promote Osteoblastogenesis and Inhibit Osteoclastogenesis against Osteoporosis via Acting on Osteoblast-Osteoclast Communication
Source: Oxid Med Cell Longev. 2023 Jan 31;2023:7212642. doi: 10.1155/2023/7212642 (PMC9904896; doi:10.1155/2023/7212642)
Supplement: Supplementary Materials — Suppl. table 1: the working parameters of 15 components by LC-MS/MS. Suppl. Figure 1: cell identification and determination of medicated serum concentrations by MTT assay. (A) Representative ALP-staining image of OBs (scale bar: 100 μm). (B) Representative TRAP staining image of OCs (scale bar: 100 μm). The cell viability of (C) OBs and (D) OCs which were cultured for 24 h or 48 h with different concentrations of (i) Co serum, (ii) EF serum, (iii) LLF serum, and (iv) EF&LLF serum. Data are represented as mean ± SEM, n = 3. ∗P < 0.05 and ∗∗P < 0.01 compared with the serum-free group. Suppl. table 2: the methods of cell grouping and administration. Suppl. Figure 2: the schematic diagram of OB-OC coculture by which we observed the indirect effect of EF&LLF on OBs. We created this schematic diagram with http://biorender.com. Suppl. Figure 3: the schematic diagram of OB-OC coculture by which we observed the indirect effect of EF&LLF on OCs. We created this schematic diagram with http://biorender.com. Suppl. table 3: the administration methods of coculture system. Suppl. table 4: the antibodies used for IF analysis. Suppl. table 5: the primers used for qPCR analysis. Suppl. table 6: the antibodies used for WB analysis. Suppl. table 7: regression equation and correlation coefficient r of standard curves of 15 components. Suppl. table 8: components content of medicated serum. Suppl. Figure 4: the cytokine expressions of OBs and OCs detected by WB. (A–D) The protein expression of TGF-β1, M-CSF, and OPG of OBs was measured by WB assay, and β-tubulin was used for normalization. (E–H) The protein expression of TGF-β1, Atp6v0d2, and BMP-2 of OCs was measured by WB assay, and β-tubulin was used for normalization. [file 7212642.f1.docx]

## Supplementary Materials

**Suppl. table 1 The working parameters of 15 components by LC-MS/MS**

| Components | Ionization mode | Q1（m/z） | Q3（m/z） | Collision voltage（V） |
| --- | --- | --- | --- | --- |
| nuezhenoside G13 | ESI^+^ | 1095.2 | 933.1 | 50 |
| oleonuezhenide | ESI^+^ | 1095.2 | 933.1 | 50 |
| epimedin A | ESI^+^ | 861.1 | 331 | 40 |
| epimedin B | ESI^+^ | 831.1 | 553 | 41 |
| epimedin C | ESI^+^ | 845.1 | 553 | 43 |
| ligustroflavone | ESI^+^ | 725.2 | 270.9 | 20 |
| specneuzhenide | ESI^+^ | 709.1 | 515 | 40 |
| icariin | ESI^+^ | 677.1 | 531 | 13 |
| icaritin | ESI^+^ | 369 | 312.9 | 26 |
| anhydroicaritin | ESI^+^ | 369 | 312.9 | 26 |
| baohuoside I | ESI^-^ | 513 | 350.9 | 28 |
| hyperoside | ESI^-^ | 462.9 | 300.9 | 20 |
| chlorogenic acid | ESI^-^ | 353 | 190.9 | 8 |
| salidroside | ESI^-^ | 299.9 | 88.8 | 10 |
| hydroxytyrosol | ESI^-^ | 152.9 | 123 | 7 |
| genistein | ESI^+^ | 271.1 | 90.9 | 36 |


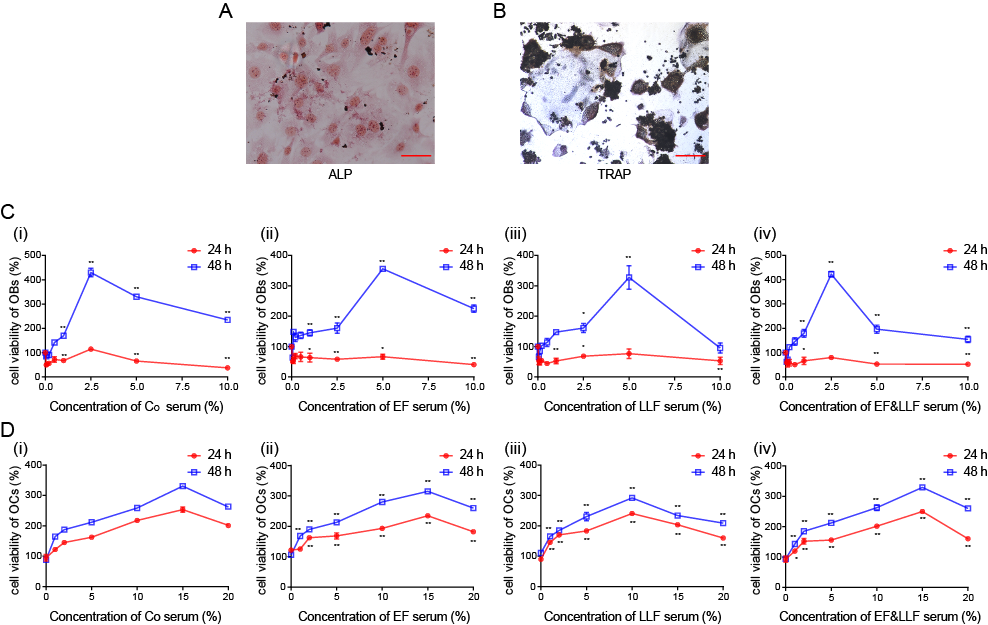


**Suppl. Fig. 1 Cell identification and determination of medicated serum concentrations by MTT assay.** (A) Representative ALP-staining image of OBs (scale bar: 100 µm). (B) Representative TRAP-staining image of OCs (scale bar: 100 µm). The cell viability of (C) OBs and (D) OCs which were cultured for 24 h or 48 h with different concentrations of (i) Co serum, (ii) EF serum, (iii) LLF serum and (iv) EF&LLF serum. Data are represented as mean ± *SEM*, *n* = 3. ^*^*P* < 0.05 and ^**^*P* < 0.01 compared with serum-free group.

**Suppl. table 2 The methods of cell grouping and administration**

| Cell type | Cell model | Group | Treatment |
| --- | --- | --- | --- |
| OBs | normal culture | Co | α-MEM CM + 2.5% Co serum |
|  |  | EF | α-MEM CM + 5% EF serum |
|  |  | LLF | α-MEM CM + 5% LLF serum |
|  |  | EF&LLF | α-MEM CM + 2.5% EF&LLF serum |
|  | TGF-β1 stimulation | TGF-β1 | α-MEM CM + 2.5% Co serum + 10 ng/mL TGF-β1 |
|  |  | TGF-β1+EF | α-MEM CM + 5% EF serum + 10 ng/mL TGF-β1 |
|  |  | TGF-β1+LLF | α-MEM CM + 5% LLF serum + 10 ng/mL TGF-β1 |
|  |  | TGF-β1+EF&LLF | α-MEM CM + 2.5% EF&LLF serum + 10 ng/mL TGF-β1 |
|  | TNF-α stimulation | TNF-α | α-MEM CM + 2.5% Co serum + 50 ng/mL TNF-α |
|  |  | TNF-α+EF | α-MEM CM + 5% EF serum + 50 ng/mL TNF-α |
|  |  | TNF-α+LLF | α-MEM CM + 5% LLF serum + 50 ng/mL TNF-α |
|  |  | TNF-α+EF&LLF | α-MEM CM + 2.5% EF&LLF serum + 50 ng/mL TNF-α |
| OCs | normal culture | Co | DMEM CM + 15% Co serum |
|  |  | EF | DMEM CM + 15% EF serum |
|  |  | LLF | DMEM CM + 10% LLF serum |
|  |  | EF&LLF | DMEM CM + 15% EF&LLF serum |
|  | RANKL stimulation | RANKL | DMEM CM + 15% Co serum + 50 ng/mL RANKL |
|  |  | RANKL+EF | DMEM CM + 15% EF serum + 50 ng/mL RANKL |
|  |  | RANKL+LLF | DMEM CM + 10% LLF serum + 50 ng/mL RANKL |
|  |  | RANKL+EF&LLF | DMEM CM + 15% EF&LLF serum + 50 ng/mL RANKL |
|  | OPG stimulation | OPG | DMEM CM + 15% Co serum + 80 ng/mL OPG |
|  |  | OPG+EF | DMEM CM + 15% EF serum + 80 ng/mL OPG |
|  |  | OPG+LLF | DMEM CM + 10% LLF serum + 80 ng/mL OPG |
|  |  | OPG+EF&LLF | DMEM CM + 15% EF&LLF serum + 80 ng/mL OPG |

**
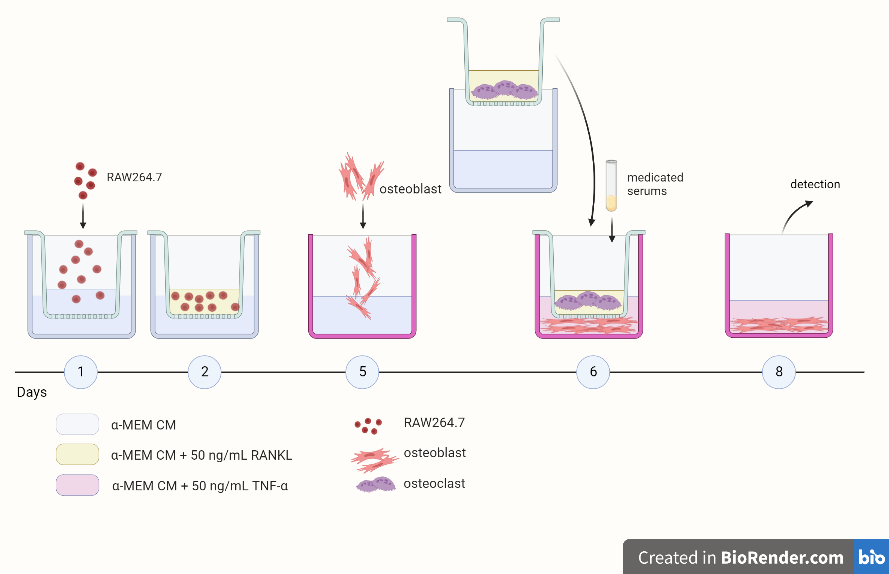
**

**Suppl. Fig. 2 The schematic diagram of OB-OC co-culture by which we observed the indirect effect of EF&LLF on OBs.** We created this schematic diagram with biorender.com.

**
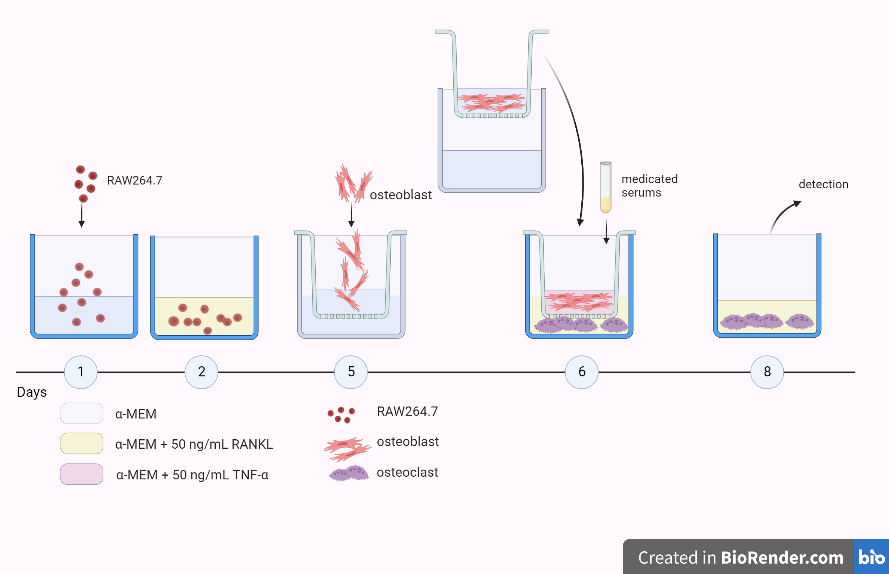
 Suppl. Fig. 3 The schematic diagram of OB-OC co-culture by which we observed the indirect effect of EF&LLF on OCs.** We created this schematic diagram with biorender.com.

**Suppl. table 3 The administration methods of co-culture system**

| The cell being tested | Group | Cell type | Position | Treatment |
| --- | --- | --- | --- | --- |
| OBs | Co | OCs | insert | α-MEM CM + 50 ng/mL RANKL + 15% Co serum |
|  |  | OBs | 24-well | α-MEM CM + 50 ng/mL TNF-α |
|  | EF | OCs | insert | α-MEM CM + 50 ng/mL RANKL + 15% EF serum |
|  |  | OBs | 24-well | α-MEM CM + 50 ng/mL TNF-α |
|  | LLF | OCs | insert | α-MEM CM + 50 ng/mL RANKL + 10% LLF serum |
|  |  | OBs | 24-well | α-MEM CM + 50 ng/mL TNF-α |
|  | EF&LLF | OCs | insert | α-MEM CM + 50 ng/mL RANKL + 10% LLF serum |
|  |  | OBs | 24-well | α-MEM CM + 50 ng/mL RANKL |
| OCs | Co | OBs | insert | α-MEM CM + 50 ng/mL TNF-α + 2.5% Co serum |
|  |  | OCs | 24-well | α-MEM CM + 50 ng/mL RANKL |
|  | EF | OBs | insert | α-MEM CM + 50 ng/mL TNF-α + 5% EF serum |
|  |  | OCs | 24-well | α-MEM CM + 50 ng/mL RANKL |
|  | LLF | OBs | insert | α-MEM CM + 50 ng/mL TNF-α + 5% LLF serum |
|  |  | OCs | 24-well | α-MEM CM + 50 ng/mL RANKL |
|  | EF&LLF | OBs | insert | α-MEM CM + 50 ng/mL TNF-α + 2.5% EF&LLF serum |
|  |  | OCs | 24-well | α-MEM CM + 50 ng/mL RANKL |

**Suppl. table 4 The antibodies used for IF analysis**

| Antibody | Description | Concentration | Manufacturer |
| --- | --- | --- | --- |
| TGF-β1 | Mouse | 1: 50 | Santa Cruz Biotechnology, Santa Cruz, USA |
| RANKL | Mouse | 1: 100 | Novus, Colorado, USA |
| M-CSF | Mouse | 1: 100 | Santa Cruz Biotechnology, Santa Cruz, USA |
| OPG | Rabbit | 1: 100 | Absin, Shanghai, China |
| ATP6v0d2 | Rabbit | 1: 100 | Absin, Shanghai, China |
| BMP-2 | Rabbit | 1: 50 | Novus, Colorado, USA |
| IGF-1 | Mouse | 1: 50 | Novus, Colorado, USA |

**Suppl. table 5 The primers used for qPCR analysis**

| Primer | Sequences of primers | Product size |
| --- | --- | --- |
| TGF-β1 | Forward: CATTGCTGTCCCGTGCAGA | 103 bp |
|  | Reverse: AGGTAACGCCAGGAATTGTTGCTA |  |
| OPG | Forward: CTCATCAGTTGGTGGGAATGAAGA | 107 bp |
|  | Reverse: ACCTGGCAGCTTTGCACAATTA |  |
| RANKL | Forward: GCAGCATCGCTCTGTTCCTGTA | 164 bp |
|  | Reverse: GCATGAGTCAGGTAGTGCTTCTGTG |  |
| M-CSF | Forward: GAATGACTGAACCTGCCTGCTGAA | 117 bp |
|  | Reverse: AGGCCAGCTCAGTGCAAGAA |  |
| Atp6v0d2 | Forward: CGAGGCATTCTACAAATTCTGCAA | 124 bp |
|  | Reverse: TTCAGTGCCAAATGAGTTCAGAGTG |  |
| IGF-1 | Forward: GCACTCTGCTTGCTCACCTTTA | 148 bp |
|  | Reverse: TCCGAATGCTGGAGCCATA |  |
| BMP-2 | Forward: ACCGTGCTCAGCTTCCATCAC | 170 bp |
|  | Reverse: CTATTTCCCAAAGCTTCCTGCATTT |  |
| β-actin | Forward: CACTTTCTACAATGAGCTGCG | 129 bp |
|  | Reverse: CTGGATGGCTACGTACATGG |  |

**Suppl. table 6 The antibodies used for WB analysis**

| Antibody | Description | Concentration | Manufacturer |
| --- | --- | --- | --- |
| TGF-β1 | Mouse | 1: 4000 | Santa Cruz Biotechnology, Santa Cruz, USA |
| M-CSF | Mouse | 1: 500 | Santa Cruz Biotechnology, Santa Cruz, USA |
| OPG | Rabbit | 1: 500 | Absin, Shanghai, China |
| ATP6v0d2 | Rabbit | 1: 500 | Absin, Shanghai, China |
| BMP-2 | Rabbit | 1: 1000 | Novus, Colorado, USA |
| β-Tubulin | Rabbit | 1: 1000 | Cell Signaling Technology, Boston, USA |

**Suppl. table 7 Regression equation and correlation coefficient r of standard curves of 15 components**

| Components | Regression equation | *r* |
| --- | --- | --- |
| nuezhenoside G13 | Y = 0.00006X - 0.0035 | 0.9976 |
| oleonuezhenide | Y = 0.00006X - 0.0015 | 0.9992 |
| epimedin A | Y = 0.0003X + 0.0275 | 0.998 |
| epimedin B | Y = 0.0002X + 0.0074 | 0.999 |
| epimedin C | Y = 0.0002X + 0.011 | 0.9992 |
| ligustroflavone | Y = 0.0017X - 0.0563 | 0.9997 |
| specneuzhenide | Y = 0.0008X - 0.0548 | 0.9986 |
| icariin | Y = 0.0089X - 0.1048 | 0.9998 |
| icaritin | Y = 0.0022X + 0.0779 | 0.9994 |
| anhydroicaritin | Y = 0.0107X - 0.9346 | 0.9979 |
| baohuoside I | Y = 0.0006X + 0.0368 | 0.9986 |
| hyperoside | Y = 0.001X - 0.0152 | 0.9978 |
| chlorogenic acid | Y = 0.0025X - 0.1597 | 0.9988 |
| salidroside | Y = 0.00004X + 0.0027 | 0.9988 |
| hydroxytyrosol | Y = 0.001X - 0.1064 | 0.9979 |

**Suppl. table 8 Components content of medicated serum**

| Components | EF&LLF (ng/mL) | EF (ng/mL) | LLF (ng/mL) |
| --- | --- | --- | --- |
| nuezhenoside G13 | 63.74± 2.97 |  | 75.72± 3.87 |
| oleonuezhenide | 48.40±12.83 |  | 48.20± 9.74 |
| epimedin A | 71.46± 6.31 | 51.38± 17.07 |  |
| epimedin B | 29.93± 5.48 | 8.86± 4.06^*^ |  |
| epimedin C | 15.38± 3.77 | 12.48± 3.92 |  |
| ligustroflavone | 46.82± 4.25 |  | 41.29± 1.63 |
| specneuzhenide | 81.74± 4.25 |  | 77.43± 2.65 |
| icariin | 23.79± 2.02 | 17.36± 0.80^*^ |  |
| icaritin | 133.04±39.65 | 527.41±130.35^*^ |  |
| anhydroicaritin | 53.02±12.73 | 42.37± 10.50 |  |
| baohuoside I | 28.28± 6.54 | 12.13± 3.77 |  |
| hyperoside | 29.56± 1.80 | 23.98± 3.94 |  |
| chlorogenic acid | 86.39± 2.47 | 74.61± 4.79 |  |
| salidroside | 387.20±63.16 |  | 159.62±28.78^*^ |
| hydroxytyrosol | 156.28±12.43 |  | 120.66± 2.83^*^ |

^*^*P* < 0.05 compared with EF&LLF group. Data are represented as mean ± *SEM*, *n* = 3.


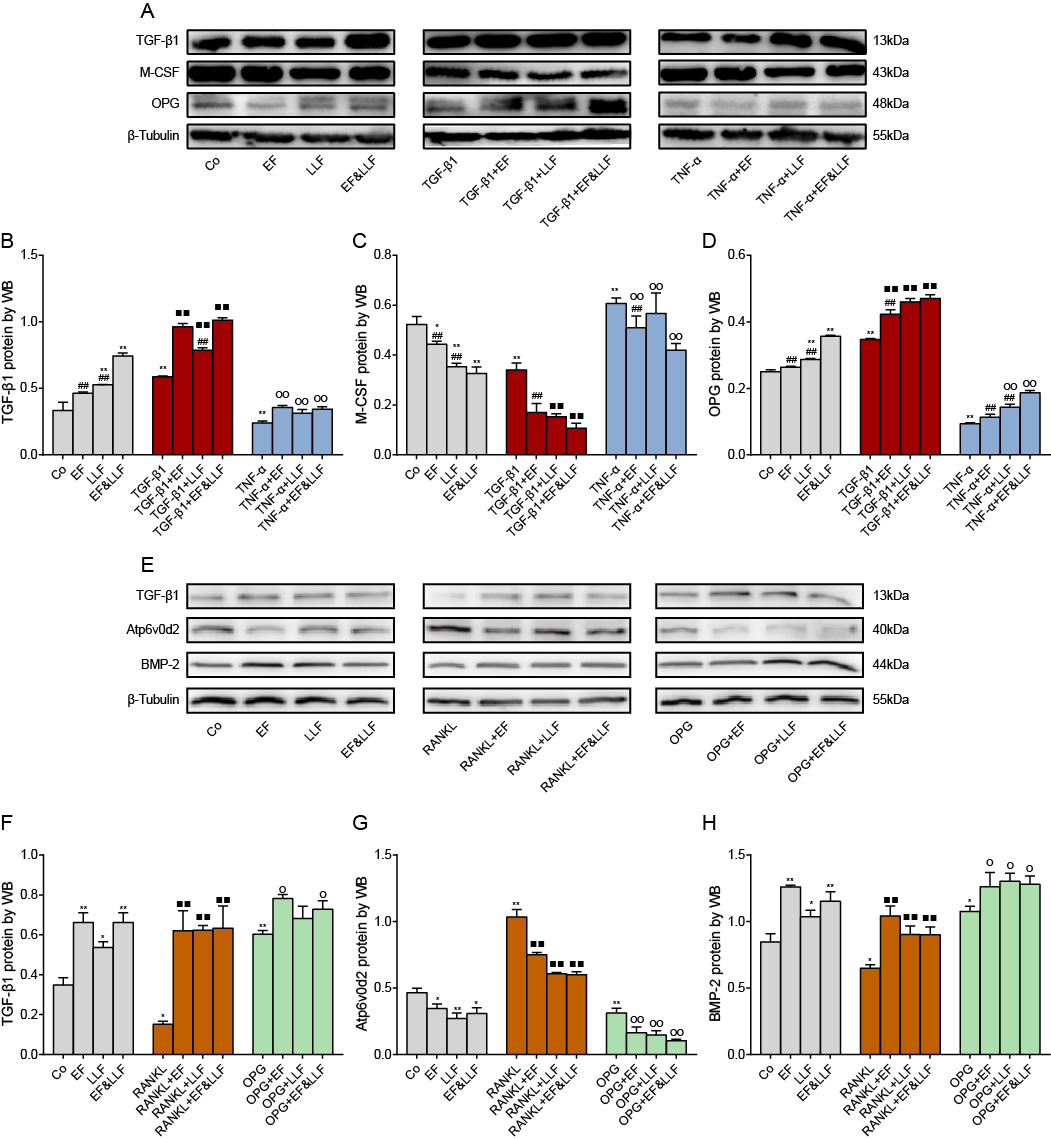


**Suppl. Fig. 4 The cytokine expressions of OBs and OCs detected by WB.** (A-D) The protein expression of TGF-β1, M-CSF and OPG of OBs was measured by WB assay, and β-Tubulin was used for normalization. (E-H) The protein expression of TGF-β1, Atp6v0d2 and BMP-2 of OCs was measured by WB assay, and β-Tubulin was used for normalization.
